# Distribution and Inferred Evolutionary Characteristics of a Chimeric ssDNA Virus Associated with Intertidal Marine Isopods

**DOI:** 10.3390/v9120361

**Published:** 2017-11-26

**Authors:** Kalia S. I. Bistolas, Ryan M. Besemer, Lars G. Rudstam, Ian Hewson

**Affiliations:** 1 Department of Microbiology, Cornell University, Ithaca, NY 14850, USA; hewson@cornell.edu; 2 New Visions Life Sciences, Boards of Cooperative Educational Services of New York State, Ithaca, NY 14850, USA; rmb1547@uncw.edu; 3 University of North Carolina at Wilmington, Wilmington, NC 28403, USA; 4 Department of Natural Resources and the Cornell Biological Field Station, Cornell University, Bridgeport, NY 14850, USA; rudstam@cornell.edu

**Keywords:** ssDNA virus, viromics, isopod, chimeric, intertidal, Cruciviridae, CRESS-DNA

## Abstract

Aquatic invertebrates are common reservoirs of a rapidly expanding group of circular Rep-encoding ssDNA (CRESS-DNA) viruses. This study identified and explored the phylogenetic relationship between novel CRESS-DNA viral genotypes associated with Pacific intertidal isopods *Idotea wosnesenskii*, *Idotea resecata*, and *Gnorimosphaeroma oregonensis*. One genotype associated with *I. wosnesenskii*, IWaV278, shared sequence similarity and genomic features with Tombusviridae (ssRNA) and Circoviridae (ssDNA) genomes and was putatively assigned to the Cruciviridae clade comprising chimeric viruses. The complete genome of IWaV278 (3478 nt) was computationally completed, validated via Sanger sequencing, and exhibited sequence conservation and codon usage patterns analogous to other members of the Cruciviridae. Viral surveillance (qPCR) indicated that this virus was temporally transient (present in 2015, but not 2017), specific to *I. wosnesenskii* at a single collection site (Washington, DC, USA), more prevalent among male specimens, and frequently detected within exoskeletal structures. 18S rRNA sequences identified two alveolate protists associated with IWaV278-positive tissues and mechanical epibiont removal of ciliated exoskeletal structures eliminated viral detection, suggesting that the putative host of IWaV278 may be an epibiont of *I. wosnesenskii*. This investigation provides additional phylogenetic evidence to resolve Cruciviridae evolution and offers insight into the biogeography, specificity, and potential host of a crucivirus genotype.

## 1. Introduction

Crustaceans are an abundant and diverse group of arthropods that populate nearly all intertidal ecosystems worldwide [1,2]. These organisms often provide consumptive control of primary productivity, enhance microbial cycling of organic material, and serve as high-value nutritional resources for consumers [3,4,5]. Despite their abundance and ecological importance, the microbial communities associated with aquatic crustaceans remain largely understudied. High-throughput metagenomic sequencing efforts have filled significant gaps in our understanding of the composition and structure of some crustacean microbial consortia. In particular, viral community profiling (i.e., viral metagenomics, or “viromics”) has revealed a plethora of novel viral genotypes that may play a role in mediating crustacean ecology [6,7,8,9,10]. These viromic surveys routinely illustrate the ubiquity and extreme genomic diversity of circular Rep-encoding single stranded (ss)DNA, or “CRESS-DNA”, viruses among aquatic invertebrates [8,10,11]. The discovery of novel CRESS-DNA viruses has revealed new phylogenetic clades with an assortment of unique genomic architectures in a range of environmental reservoirs and hosts, illuminating the richness and global pervasiveness of ssDNA viruses [12,13,14]. This study builds upon viral discovery efforts to characterize ssDNA viral genomes in natural ecosystems, ultimately filling key gaps in our understanding of the distribution and ecology of CRESS-DNA viruses associated with ecologically relevant crustacean mesograzers.

CRESS-DNA viruses are non-enveloped, icosahedral particles containing small (<6 kb), monopartite, circular genomes which comprise, at minimum, one structural gene encoding a capsid protein (Cp) and one nonstructural gene encoding a rolling circle replication initiator protein (Rep; [12]). These viruses share genomic features and sequence similarity to well characterized eukaryote-associated ssDNA viruses from families Circoviridae, Geminiviridae, and Nanoviridae [12]. Characterization of representative (pathogenic) members of these viral families suggest that CRESS-DNA viruses exhibit high nucleotide substitution rates comparable to those of RNA viruses (10^−3^ to 10^−4^ substitutions site^−1^ year^−1^; [15]), and display a proclivity for genomic recombination within and between clades [16,17,18]. These two characteristics, in combination with other adaptive mechanisms, such as reassortment or gene duplication, are likely responsible for the observed richness of CRESS-DNA viruses among putative invertebrate hosts. ssDNA viruses may also recombine across evolutionarily distinct groups of viruses, leading to “hybrid”, or “chimeric” viruses encoding Tombusviridae-like capsid proteins (homologous to positive-sense ssRNA viruses), and Circo-, Nano-, and Geminivirus-like replication initiator proteins (homologous to ssDNA viruses) on the same monopartite genome [16,18,19,20,21,22]. These chimeric genomes, tentatively classified as “cruciviruses”, were initially identified in 2012 in Boiling Springs Lake, CA, USA (Boiling Springs Lake RNA-DNA hybrid virus, BSL-RDHV; [19]) and have since been detected in viromes associated with marine and peatland ecosystems [21], sewage treatment reservoirs and other vertebrate fecal samples [20], proprietary nucleic acid extraction spin columns [16], and arthropods [9,23,24,25], among others [22,26]. The discovery of novel chimeric genomes convolutes existing viral cladistics, but provides an opportunity to better understand how genetic mechanisms such as recombination contribute to total viral diversity [22,27]. These newly-discovered ssDNA viral genomes, including those reported in this investigation, also indicate that a range of previously unexplored niches, including aquatic crustaceans, may serve as prime sites for viral evolution.

Isopods are among the most broadly distributed and diverse order of crustaceans, with over 6000 characterized aquatic species that exploit a range of ecological strategies, from parasitism to filter feeding [28]. This study examined isopods of families Idoteidae and Sphaeromatidae (order: Isopoda), principal degraders of kelp rafts (*Nereocystis sp.*) and detrital biomass in littoral ecosystems on the Pacific Coast of North America [29,30,31,32], to determine if these ecologically important mezograzers harbor evolutionarily significant ssDNA viral genotypes. Viromic sequencing of the viral consortia of *Idotea* (*Pentidotea*) *wosnesenskii*, *Idotea* (*Pentidotea*) *resecata*, and *Gnorimosphaeroma oregonensis* illustrated that these organisms may function as reservoirs for CRESS-DNA viruses, including chimeric genomes associated with the Cruciviridae. This investigation aimed to integrate high throughput sequencing, viral quantitation (qPCR), and invertebrate biogeography/anatomy to describe (1) the genomic architecture; (2) codon usage patterns; (3) genotype distribution; and (4) tissue specificity of the chimeric CRESS-DNA virus, IWaV278, associated with *I. wosnesenskii*.

## 2. Materials and Methods 

### 2.1. Isopod Collection and Virome Analysis 

Marine isopods *Idotea (Pentidotea) wosnesenskii, Idotea (Pentidotea) resecata* (Family: *Idoteidae*), and *Gnorimosphaeroma oregonensis* (Family: *Sphaeromatidae*) were collected from littoral ecosystems on the Pacific coast of North America between September 2015 and April 2017 and preserved at −80 °C (Table S1). Five (*Idotea spp.*) or 10 (*G. oregonensis*) isopods per species were gently rinsed with 0.02 μm-filtered phosphate-buffered saline (PBS) with closed opercula to remove sand and coarse particles and homogenized for 10 min (2.0 mm BashingBead™ Lysis Tubes, Zymo Research, Irvine, CA, USA) prior to viral purification. Viromic libraries were prepared via established methods to purify and enrich for small, circular, ssDNA molecules, including CRESS-DNA virus genomes [33]. Briefly, homogenates were pooled, 0.2 μm syringe filtered to reduce cellular contamination, and concentrated via polyethylene glycol (PEG) precipitation. Specific protocols are detailed in [6]. Resuspended concentrates were enzymatically digested with nucleases to exclude non-encapsidated nucleic acids prior to DNA extraction using the ZR viral extraction kit (Zymo Research, Irvine, CA, USA). Extractions were enriched for circular ssDNA templates using isothermal rolling circle amplification (Genomiphi Whole Genome Amplification Kit, GE Healthcare, Little Chalfont, UK), confirmed by PicoGreen incorporation and gel electrophoresis. DNA was then fragmented and barcoded via the Nextera XT DNA Library Preparation Kit (Illumina, San Diego, CA, USA) prior to 2 × 250 bp paired-end Illumina MiSeq sequencing at Cornell University Core Laboratories Center (Ithaca, NY, USA). Virome libraries are associated with Genbank accession numbers SAMN07716012-SAMN07716014 (BioProject PRJNA412272).

Resulting reads were trimmed for quality/size and de novo assembled using a de Bruijn algorithm in CLC workbench (v.8.5.1, Qiagen, Hilden, Germany) with parameters described in [6]. Sequencing generated 10,617,454 cumulative reads (10,539,144 reads after quality trimming), which assembled into 39,074 contigs with an average N50 of 1310 nt (Table S1). Assembled sequences (contigs) were compared to a locally-curated database of known laboratory artifacts derived from the high-throughput sequencing preparation pipeline (BLASTn, e-value < 1 × 10^−5^; [6,34]), and potential artifact sequences were excluded from further investigation. CRESS-DNA and chimeric viruses were identified via BLASTx against the NCBI non-redundant database (e-value < 1 × 10^−5^). Open reading frames (ORFs) of putatively novel genomes were demarcated by GetORF v.6.6 (ORFs > 300 nt; EMBOSS, http://emboss.sourceforge.net/apps/cvs/emboss/apps/getorf.html) and annotated via BLASTx against the non-redundant database (e-value < 1 × 10^−5^; [34]). Predicted ORFs associated with genome replication (Rep amino acid sequences) were aligned in MUSCLE v.3.8 [35], manually masked, and assessed for phylogenetic relationship to best BLASTx hits. Maximum likelihood phylogenies were constructed using SMS (smart model selection) implemented in PhyML [36] and visualized in FigTree v.1.4.2. (http://tree.bio.ed.ac.uk/software/figtree/) Branch support was determined by approximate likelihood ratio testing (SH-like aLRT; [37]).

Codon usage biases were evaluated using CodonW v.1.4.4 (http://codonw.sourceforge.net/) and webservers CAIcal (genomes.urv.es/CAIcal/) and compseq (emboss.bioinformatics.nl/cgi-bin/emboss/compseq). Metrics included: (1) contig-wide nucleotide composition (total %GC); (2) GC content at codon sites (%GC1, %GC2, and %GC3) depicting relative GC preference at non-synonymous sites; (3) effective numbers of codons (ENC) denoting the degree of codon bias, where 20 denotes single codon usage per amino acid and 61 denotes random codon usage; (4) relative synonymous codon usage (RSCU), indicating over- or under-utilization (RSCU > 1.6 or RSCU < 0.6, respectively) of AT- or GC-terminating codons; (5) codon adaptation indices (CAI) describing the degree of relatedness of codon usage patterns of a query sequence to a set of reference genes; and (6) dinucleotide distributions describing the observed frequency of CpG sites standardized to expected frequency of CpG sites.

### 2.2. IWaV278 Genome Analysis and Quantitation

Among contigs sharing sequence similarity to CRESS-DNA viruses, chimeric genotype IWaV278 recruited the greatest number of reads nt^−1^ among isopod libraries and contained the minimum ORFs required for viral viability (both Rep and Cp) and was, therefore, selected for further investigation. IWaV278 was assessed for a nonanucleotide origin of replication (ori: NANTATTAC) and associated stem loop (Mfold Web Server; [38]), coverage (80% similarity over 50% of read length), presence of structural Cp domains characteristic of Tombusviridae genera, and presence of canonical CRESS-DNA virus rolling circle replication motifs (rolling circle replication motifs I-III, or superfamily 3 helicase motifs Walker A/B and Motif C) via CLC workbench v.8.5.1 (Qiagen, Hilden, Germany). IWaV278 was completed and confirmed through a combination of de novo computational assembly (CLC workbench v.8.5.1) and inverse PCR. Following initial genome assembly and computational validation, IWaV278 amplicons were generated from polymerase chain reaction (PCR) using outward-bound primers (primer and reaction parameters detailed in Table S2). Amplicons were gel purified (Zymo Research, Irvine, CA, USA), cloned (pGEM-T vector, Promega, Madison, WI, USA and JM109 competent *E. coli*, Invitrogen, Carlsbad, CA, USA), and recovered (Zyppy^TM^ Plasmid Miniprep Kit, Zymo Research, Irvine, CA, USA) prior to Sanger sequencing at Cornell University Core Laboratories Center (Ithaca, NY, USA) to confirm computational circularization.

Codon usage metrics (%GC1-3, ENC, RSCU, CAI, and dinucleotide distributions defined in Section 2.1) of IWaV278-*rep* and IWaV278-*cp* were assessed via the software described above and compared to codon usage patterns in a set of reference genes, including: chimeric virus replication ORFs (*rep*), chimeric virus capsid/coat ORFs (*cp*), Tombusviridae capsid/coat ORFs (*cp*), and metazoan-associated CRESS-DNA virus replication ORFs (*rep*). Nucleotide sequences for reference genes were curated via NCBI search of complete coding sequences using virus family and gene names (e.g., “Circoviridae” + “*rep*”), parsed to include complete ORFs without internal stop codons, and reported in File S1 and Table S6.

Prevalence and load of IWaV278 were assessed in DNA extractions from whole and sub-dissected isopods (Tissue-Insect Extraction Kit, Zymo Research, Irvine, CA, USA) via quantitative PCR (qPCR). *I. wosnesenskii* were classified as juvenile, female, or male by size (juvenile; <8 mm) and the presence of a characteristic stylet and genital papillae (penes; male) or marsupium (female; [39]). To evaluate viral presence in isolated organ systems, adult *I. wosnesenskii* were aseptically dissected in 70% ethanol using sterile forceps and iris scissors prior to DNA extraction. IWaV278 load was also quantitated for washed and unwashed pleopod tissue to determine the impact of putative epibiont presence on viral detection. Dissected pleopod pairs from single isopods were separated, and half of the tissue was vortexed for 5 min in 250 μL nuclease free H_2_O. Tissues were then transferred into a new sterile tube and washes were repeated two additional times using 250 μL nuclease free H_2_O. Single pleopods from unwashed and washed subdissections were stained with 4′,6-diamidino-2-phenylindole dihydrochloride (DAPI) and visualized via fluorescent microscopy to confirm absence of unicellular organisms on pleopod cilia/setae (Figure 5). DNA was extracted from unwashed tissue, washed tissue, and wash supernatant via a tissue-insect extraction kit (Zymo Research, Irvine, CA, USA) and assessed for IWaV278 load. The identity of unicellular epibionts was evaluated in wash supernatant using universal primers EU347F and EU929R which target eukaryotic 18S rRNA regions V3-V4 (~582 bp; Table S2). Amplicons were gel purified, cloned, and sequenced via the method described above. Resulting sequences were annotated via BLASTn (e-value < 1 × 10^−5^, [34]).

qPCR primers and probes were designed via Primer3 [40] to target IWaV278-*rep*. Reaction conditions and primer/probe/standard sequences are detailed in Table S2. Valid reactions were defined by reaction efficiency (>90%), standard regression linearity (R^2^ > 0.98), and no detection in negative controls. The lower limit of detection of IWaV278 corresponded to 39.9 standard copies μL^−1^ (average Ct: 37.96). All qPCR reactions were assessed in duplicate on a StepOnePlus^TM^ Real-Time PCR system (Applied Biosystems, Foster City, CA, USA) with eight-fold standard dilutions. Samples were re-assessed if the Ct standard deviation between technical replicates was > 0.5. Sterile (0.02 μm filtered, nuclease-free) H_2_O was concurrently processed with experimental samples as negative controls [16,41]. IWaV278-*rep* was not detected in any control samples (*n* = 6), indicating that this genotype is likely not associated with extraction spin columns or reagents. Corrected copy number of IWaV278-*rep* amplicons were interpolated from a standard curve (StepOnePlus software v.2.3, Foster City, CA, USA), adjusted for extraction, elution, and reaction dilution volumes, and standardized by animal or dissection wet weight. IWaV278 prevalence and load were defined as the total frequency of positive detection among specimens and mean copy number g^−1^ wet weight between duplicates among positive specimens, respectively. Raw Ct values can be accessed via Table S5.

## 3. Results and Discussion

### 3.1. Identification of Isopod-Associated CRESS-DNA and Chimeric Viruses

Viromes from three temperate, littoral isopods were enriched for small, circular ssDNA templates and cumulatively generated 39,074 de novo assembled contigs. Congruent with viromes from other marine or metazoan systems, 48.6–55.1% of resulting contigs per library could not be annotated (Table S1; [42,43,44,45]). Among remaining contigs, 29 contigs exhibited sequence similarity to known metazoan-associated CRESS-DNA virus genomes (BLASTx e-value < 1 × 10^–5^; Table S3). Despite template enrichment via rolling circle amplification [46] and loose read recruitment parameters (80% identity over 50% of read length), the majority of these contigs did not represent numerically-significant components of isopod viromes (Table S3). Putative rolling circle replication (Rep) ORFs of CRESS-DNA virus-like contigs were taxonomically variable (Figure S1) and did not exhibit significant or directional variation in dinucleotide frequency or codon usage between viruses associated with sympatric isopod genera, though total %GC content (and therefore CpG and GC3 composition) was marginally higher among *rep* ORFs associated with *I. wosnesenskii* (Table S4; Figure S2).

Low genotype coverage and nonspecific taxonomic/genomic diversity may indicate that most novel CRESS-DNA viruses identified in isopod viromes are transiently associated with crustaceans and do not partake in active replication within metazoan tissues. However, one genome, IWaV278 (*I. wosnesenskii* associated viral contig-278), recruited the greatest number of reads (nt^−1^) among ssDNA viral contigs, contained the minimum ORFs required for viral viability (Rep and Cp), and exhibited unique homology to divergent viral families. Therefore, investigation of IWaV278 allows insight into the ecological and evolutionary dynamics of a putatively chimeric virus identified in a natural ecosystem.

**Figure 1:**
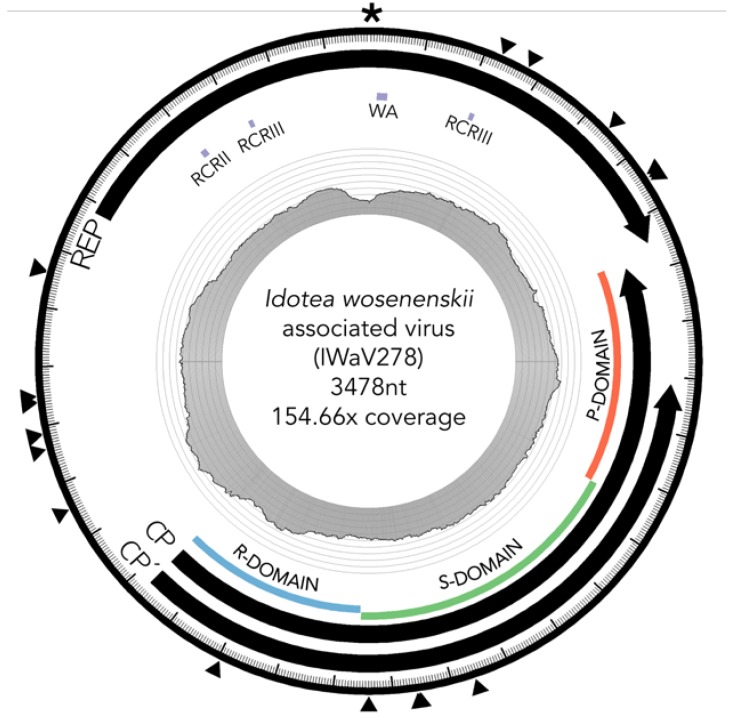
Diagram of IWaV278 genome. Scaled depiction of chimeric virus IWaV278 exhibiting type III genomic architecture [12]. Annuli from the center: (i) line graph represents coverage per site; (ii) grey tiles indicate the position of rolling circle Rep motifs RCRII (LHLQG) and RCRIII (YCRK and YALK), Rep-associated Walker-A motif (WA: GSTGTGKS), and Cp domains R (RNA/genome-interacting), S (shell), and P (protruding); (iii) black arrows denote Rep, Cp, and Cp’ ORF size, and orientation; (iv) external arrows indicate substitution sites; (v) the asterisk denotes position of the putative ori (nonanucleotide motif: TAATATTAC). Genome visualized via Circos (v.0.69-3; [47]).

Viral genotype IWaV278, contained a capsid ORF (Cp) homologous to those found in positive sense ssRNA viruses of family Tombusviridae, and a rolling circle replication ORF (Rep) homologous to those commonly observed in ssDNA viruses of family Circoviridae (Figure 1; [22]). Consequently, IWaV278 likely represents a novel member of the proposed Cruciviridae clade [21], which comprises other chimeric genomes that share sequence similarity to both ssRNA and ssDNA viral genomes. The identification of this chimeric genotype reiterates the inadequacy of short-read based taxonomic assignment and highlights the challenges associated with whole genome-based approaches to viral phylogenomics. 

### 3.2. Characterization of I. wosnesenskii Associated Chimeric Virus IWaV278 

IWaV278 was characterized via computational assembly (de Bruijn) and Sanger sequencing (inverse PCR, accession number: MG023125). The IWaV278 genome is larger than most CRESS-DNA viruses (3478 nt), but comparable in size to members of the Cruciviridae/Tombusviridae clades [16,21,22]. The genome had approximately 154.7× coverage (2450 total reads) in *I. wosnesenskii* viromes enriched for circular, ssDNA templates. This genotype is complete (circularized), and displays the general genome architecture of type III CRESS-DNA viruses (Figure 1; [12]). IWaV278 contains a 728 nt intergenic region with two flanking ambisense ORFs: (1) a 385AA (1245 nt) sense-oriented rolling circle replication (Rep) ORF homologous to the replicase gene of *Tadarida brasiliensis* circovirus genotype 1 (YP_009170674.1, e-value < 2 × 10^−28^), and (2) a 418AA (1503 nt) antisense-oriented putative structural ORF (Cp) homologous to the predicted capsid protein of Cruciviridae genotype CRUV-15-B (AQU11701.1, e-value < 9 × 10^−29^) with an alternate stop codon that truncates 83AA (243 nt) from the C-terminal domain of Cp (Cp’).

#### 3.2.1. Replication Initiation ORF (IWaV278-Rep)

**Figure 2:**
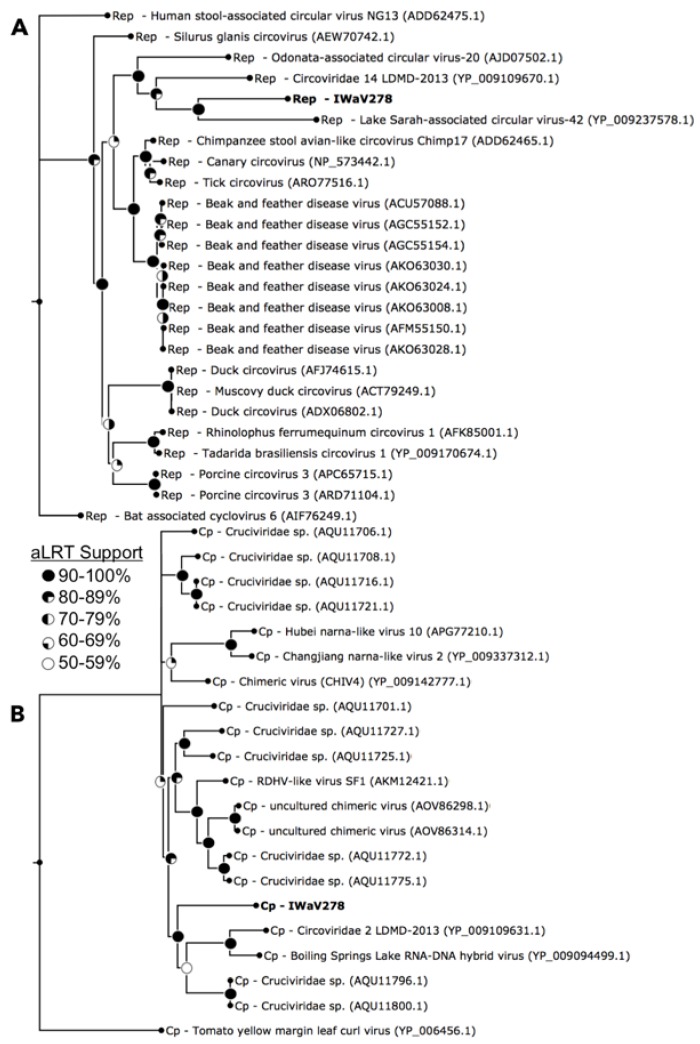
Maximum likelihood phylogeny of Rep (**A**) and Cp (**B**) open reading frames. Phylogenies were constructed via smart model selection (Rep: LG+G+I, Cp: RtREV+G+F) implemented in PhyML and visualized via FigTree v.1.4.2. Terminal nodes indicate IWaV278-ORFs and associated best BLASTx hits (e-value <1 × 10^−5^). Sequences and outgroups (Rep: bat associated cyclovirus 6; Cp: tomato yellow margin leaf curl virus) were aligned in MUSCLE v.3.8 and manually masked (Rep: 306AA, Cp: 523AA). Internal nodes represent SH-like aLRT branch support (nodes exhibiting <50% aLRT support collapsed; [37]).

IWaV278-Rep contained characteristic features of the origin of replication (ori) of Circoviridae genomes, including a canonical nonanucleotide motif (TAATATTAC) enclosed in a stem loop (ΔG = −6.19 kcal/mol; Mfold; [38]; Figure S3). IWaV278-Rep also encoded rolling circle replication motifs II (RCRII: LHLQG) and III (RCRIII: YCRK/YALK), and a superfamily 3 helicase (S3H) Walker-A motif (GSTGTGKS), which are signature features of CRESS-DNA virus Rep proteins (Figure 1). Like other reported chimeric viruses [16], IWaV278 RCR and S3H motifs were analogous to, and may be derived from, Circo-, Nano-, or Geminiviridae motifs. However, the complete nucleotide sequence of *rep* indicated overall similarity to a circovirus genome (Figure 2, Supplementary File 1, accession number: MG023125). Therefore, we speculate that IWaV278 does not exhibit evidence of intra-gene chimerism (i.e., multiple incidences of recombination within Rep) or partial Rep gene replacement, unlike several previously reported Cruciviridae genomes [16]. Phylogenetic analysis supports this hypothesis, as IWaV278-Rep associated with a monophyletic clade of non-chimeric circovirus Rep ORFs, potentially corroborating singular acquisition or complete replacement of Rep (Figure 2; [16,22]). Additionally, codon adaptation indices (CAI) indicated that codon usage patterns in IWaV278-*rep* were more common to patterns in other CRESS-DNA and chimeric virus *rep* genes (Figure S4E), relative to those in IWaV278-*cp*, providing evidence of purifying selective pressure on Rep or a recent gene acquisition event among ssDNA and ssRNA viral genomes.

#### 3.2.2. Structural ORF (IWaV278-Cp)

The predicted structural ORFs of chimeric viruses (Cp) are typically homologous to Tombusviridae capsid genes associated with economically important crop species (tomatoes, lettuce, peppers, etc.; [48]), or unclassified Nodavirus-like *Plasmopara halstedii-A* (PhV-A) and *Sclerophthora macrospora* (SmV-A) viruses associated with oomycetes [19,49,50]. *Tombusvirus, nodavirus, and crucivirus* capsids typically contain domains R (RNA/genome-interacting, interior facing), S (shell), and P (protruding, exterior facing), putatively involved in viral genome interaction, capsid composition, and host interaction, respectively. All three domains were identified within IWaV278-Cp via protein search and alignment against Pfam v.31.0 (http://pfam.xfam.org/) and InterPro v.64.0 (https://www.ebi.ac.uk/interpro/) databases, and corroborated by HHpred prediction of remotely homologous structures (Figure 1). An alternative stop codon potentially truncates the C-terminal region of the P-domain, and further transcriptomic/proteomic analysis is required to determine if this peptide is utilized as an additional structural unit. IWaV278-Cp was phylogenetically related to other chimeric virus Cp sequences from aquatic ecosystems (lakes, hot springs, and peatlands, Figure 2; [19,21,26]), potentially inferring that Cp acquisition from ssRNA genomes is a rare occurrence among ssDNA viruses, as proposed by [22]. However, in contrast to previously reported phylogenies, Cruciviridae structural genes did not form a monophyletic clade, and included capsid sequences from newly described non-chimeric, invertebrate-associated, ssRNA viruses identified among invertebrate taxa (Figure 2). These viral genomes, Hubei narna-like virus 10 and Changjiang narna-like virus 2, are putatively associated with superphylum Lophotrochozoa and subphylum Crustacea, respectively, and have both been found as endogenous elements (EVEs) in arthropod genomes [25]. This polyphyletic relationship may provide an additional link between chimeric and ssRNA genomes or imply structural convergence among ssDNA viruses associated with invertebrates.

### 3.3. IWaV278 Codon Usage Biases and Sequence Conservation

Measures of codon usage indicated that IWaV278 was unusually GC-rich among chimeric viruses (GC:AT content, Figure S4A), which was reflected in elevated CpG frequency (Figure S4F) and %GC content at synonymous sites (relative synonymous codon usage/RSCU and %GC3, Figure S4C and B, respectively). These distinct IWaV278 codon usage biases and overall greater %GC content in non-chimeric or incomplete *I. wosnesenskii*-associated CRESS-DNA virus contigs (Table S4, Figure S2) may signify that host- or habitat-specific selective pressures play a substantial role in the accumulation of synonymous substitutions among CRESS-DNA viruses associated with *I. wosnesenskii* [51]. 

Additionally, as noted by Roux et al. [22], chimeric genomes are unique among CRESS-DNA viruses in that structural ORFs (Cp) are often as conserved as replication ORFs (Rep), rather than exhibiting elevated levels of genetic divergence (<40–60% sequence similarity; [10,12]). Preliminary single nucleotide variant (SNV) sites were detected via a multinomial model for low frequency variant calling that employed read-recruitment from *I. wosnesenskii* viromes (90% similarity, 80% read length; CLC Genomics Workbench v.8.5.1). IWaV278-*rep* and IWaV278-*cp* harbored roughly equivalent quantities of predicted variable sites standardized by total ORF length, whereas IWaV278-*rep* exhibited significantly greater frequency of SNVs within these sites (*p* < 1 × 10^−2^, paired *t*-test; Figure S5). While most CRESS-DNA viruses demonstrate greater sequence conservation in *rep* than in *cp*, this finding was on par with the observed divergence in *rep* exhibited by other chimeric viruses [16,22]. The 728 nt intergenic regions (IWaV278-IR) exhibited significantly greater densities of variable sites (*p* < 1 × 10^−7^, paired *t*-test; Figure S5), relative to IWaV278 ORFs, providing further evidence of purifying selection within IWaV278-*rep* and IWaV278-*cp* ORFs. Curiously, all single nucleotide variants (SNVs) resulted in non-synonymous substitutions in IWaV278-*rep* and IWaV278-*cp* ORFs. Furthermore, within *cp*, SNVs occur predominantly within the R- and S-domains, which may play a role in genome interaction or capsid composition. Therefore, while codon usage biases and SNV sites indicate that purifying selection is exerted on the IWaV278 genome, we speculate that IWaV278 and other chimeric viruses are also subject to adaptation at specific functional sites as a consequence of recombination/gene-acquisition driven host- or habitat-specific adaptation.

### 3.4. Biogeography and Transience of IWaV278

**Figure 3:**
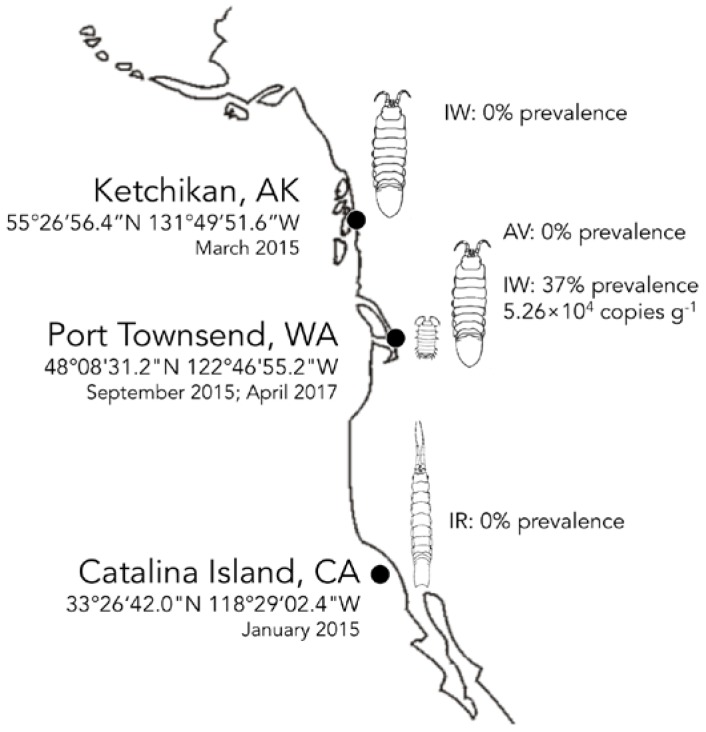
Biogeography of IWaV278 among intertidal isopods on the Pacific Coast of North America. Distribution (prevalence and average load g^−1^) of genotype IWaV278 was quantified in *Idotea wosnesenskii* from Washington and Alaska, USA, via qPCR targeting IWaV278-*rep*. IWaV278 was not detected in taxonomically-related isopods (IR—*Idotea resecata*) from the Channel Islands (USC Wrigley Institute, CA, USA), sympatric isopods (GO—*Gnorimosphaeroma oregonensis*) or primary dietary substrate (*Nereocystis* sp.).

IWaV278 was detected via qPCR in 36.7% of a single population of *I. wosnesenskii* at an average load of 5.26 × 10^4^ ± 1.40 × 10^4^ genome copies g^−1^ (wet weight; Figure 3, Table S5). Despite the wide geographic range of *I. wosnesenskii* (Alaska to Central California, USA; [39]), IWaV278 was exclusively identified at a single site (Port Townsend, WA, USA), and was absent from a northern site (Ketchikan, AK, USA; *n* = 30), indicating that dispersal of this genotype may be geographically limited by factors other than *I. wosnesenskii* distribution. IWaV278 was not detected in the primary dietary substrate of *I. wosnesenskii* (*Nereocystis sp.* kelp racks), or in genetically divergent isopods, *Gnorimosphaeroma oregonensis*, which shared an overlapping intertidal niche with IWaV278-positive *I. wosnesenskii* populations (*n* = 10). Additionally, IWaV278 was not detected in *I. resecata* (*n* = 10), a taxonomically similar isopod from the Channel Islands (USC Wrigley Institute, CA, USA), further establishing the site- and species-specificity of this genotype. IWaV278 is likely a transient virus, as there was no detection in specimens collected two years later (April 2017, *n* = 10) from previously IWaV278-positive populations. However, it remains unclear if the absence of IWaV278 in *I. wosnesenskii* collected in 2017 is due to local extirpation of the virus/alternative host or rapid viral evolution, as observed in other ssDNA viruses (10^−3^ to 10^−4^ substitutions site^−1^ year^−1^; [15]).

**Figure 4:**
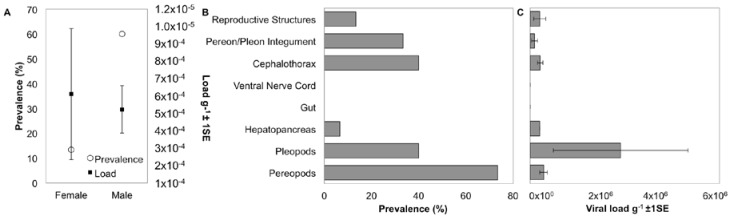
Distribution and tissue specificity of IWaV278. (**A**) Prevalence and average load g^−1^ (±1 SE) of IWaV278 in male and female *I. wosnesenskii* from the Port Townsend (WA, USA); (**B**) prevalence of IWaV278 in major *I. wosnesenskii* organ systems; and (**C**) average load g^−1^ (±1 SE) of IWaV278 in major *I. wosnesenskii* organ systems detected via qPCR targeting IWaV278-*rep*.

Prevalence of IWaV278 varied between isopod sexes, with male specimens exhibiting a significantly greater frequency of detection (60%; *n* = 15) relative to females (13.3%; *n* = 15; Pearson’s Chi-squared test with Yates’ continuity correction, *X* = 5.17, *df* = 1, *p*-value = 0.02; Figure 4). However, among IWaV278-positive specimens, viral load (g^−1^) was variable and did not differ substantially between isopod sexes (paired *t*-test; Figure 4). Furthermore, prevalence and load of IWaV278 did not vary by organism length or weight when grouped by sex and gravid females did not exhibit distinctive patterns of viral presence when compared to non-gravid or spent females (Figure S6).

### 3.5. Localization and Predicted Host of IWaV278

To date, the host and tissue tropism of most chimeric viruses remain speculative. *I. wosnesenskii* dissection and qPCR analysis indicated that the dominant virus-bearing tissues included those with chitinous integument (exoskeleton). For example, IWaV278 was consistently detected in pereopods (73.3%), pleopods (40.0%), cephalothorax (40.0%), and pereon/pleon integument (33.3%, Figure 4, Figure S7). Viral load was significantly greater in pleopod tissue relative to other tissue types, potentially indicating narrow tissue distribution (one-way ANOVA *F* = 2.79, *p* = 0.04; post hoc: Tukey multiple comparison of means, *p* < 0.05; Figure 4). Notably, IWaV278 was rarely detected in gut or hepatopancreas dissections, indicating that dietary acquisition of IWaV278 is unlikely. In contrast to many commercially-relevant metazoan-associated CRESS-DNA viruses (e.g., porcine circovirus), IWaV278 was not consistently detected in reproductive tissues, including male genital papillae (penes), male stylets, or female marsupia. IWaV278 also exhibited low prevalence and negligible load in ova, larvae, and mancae.

**Figure 5:**
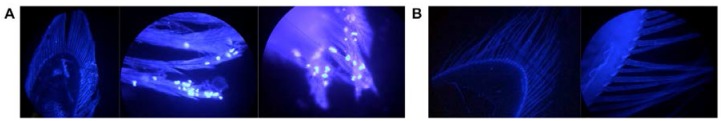
Fluorescent microscopy of *I. wosnesenskii* pleopods (40× magnification). (**A**) 4′,6-Diamidino-2-phenylindole dihydrochloride (DAPI) stained unwashed, ciliated pleopods and (**B**) pleopods washed 3× with sterile, virus-free water depicting the presence of putative epibionts and effectiveness of iterative washes in achieving epibiont removal.

Tissue types with the greatest viral prevalence and load were frequently highly ciliated (high density of bristle-like setae), particularly in male *I. wosnesenskii*. Multiple isopod genera are known to harbor a variety of photosynthetic and non-photosynthetic eukaryotic epibionts, often on analogous ciliated structures [52,53,54]. Fluorescent microscopy of *I. wosnesenskii* revealed possible unicellular organisms accumulated on pleopod cilia/setae, which could conceivably serve as cellular hosts for IWaV278 (Figure 5). IWaV278 was absent from pleopods washed with sterile, virus-free water, but detectable in the resulting wash supernatant (40% prevalence, 3.2 × 10^4^ ± 5.0 × 10^3^ copies μL^−1^ of supernatant), signifying a tenuous correlation between IWaV278 and the presence of unicellular epibionts. This observation aligns with hypotheses from previous investigations, which speculate that chimeric viruses may be associated with unicellular eukaryotic hosts [22].

To determine the identity of possible epibionts associated with *I. wosnesenskii*, we amplified and sequenced 18S rRNA from virus-positive wash supernatant. The majority of 18S rRNA sequences (88%) amplified from IWaV278-positive pleopod wash supernatant were identified as alveolates, including members of the Apicomplexa and Ciliophora (accession numbers MG023100-MG023124). In total, 11 of 25 sequences shared significant sequence similarity (BLASTn: 99–100% query cover, e-value < 1 × 10^−40^) to members of phylum Apicomplexa, 82% of which were identified as *Cephaloidophora* cf. *communis*, a marine eugregarine known to adhere to crustacean hosts (*Balanus balanus*; [55]). 11 of 25 sequences were homologous to members of phylum Ciliophora, and 73% were affiliated with the ciliate genus *Isochona*, which are sessile, ciliated, chonotrich ectosymbionts known to associate with a range of crustaceans [56]. Of the remaining 18S sequences (*n* = 3), two were identified as crustaceans, and one was identified as the chlorophyte *Ulva linza.*

We propose protozoan taxa, such as *Cephaloidophora* or *Isochona,* may function as a cellular host for IWaV278 and other chimeric viruses. Previous reports support the hypothesis that unicellular eukaryotes may serve as cellular hosts for chimeric viruses. For example, the first described chimeric genome (BSL-RDHV; [19]) was identified from Boiling Springs Lake, a highly oligotrophic, geothermally active, and acidic habitat, which restricted cellular communities to exclusively microbial taxa. Due to this unique geochemical niche, it follows that BSL-RDHV may affiliate with one or multiple of the available unicellular eukaryotic hosts (e.g., chlorophytes, stramenopiles, Euamoebida; [19]). Chimeric genotypes similar to BSL-RDHV have been predominantly identified in aquatic metagenomes, including those enriched with photosynthetic unicellular algae [21,22,23,26]. Other chimeric genomes have been unexpectedly recovered from spin columns of proprietary DNA extraction kits [16], and may be associated with the component diatomacous silica, implying a correlation between photosynthetic unicellular eukaryotes and the presence of chimeric viruses. Conservation between IWaV278-Cp (S- and P-domains) and Tombusviridae capsid genes, despite the profoundly different ecosystems in which these viruses are detected, may denote that the shell (S) and protruding (P) structural units are key viral tropogens. Consequently, the specificity and biogeography of IWaV278 may be dependent upon the distribution of associated epibionts. However, additional viral localization and microscopy is required to confirm the host of chimeric viruses.

## 4. Conclusions

Chimeric ssDNA viral genotype, IWaV278, was exclusively detected in one population of *I. wosnesenskii* at a single time point and was not identified in sympatric or taxonomically similar species, indicating that chimeric viruses may be transient or not associated with metazoans. Epibiont removal experiments indicate that the biogeography of IWaV278 may be dependent upon the distribution of unicellular protozoans among isopods (apicomplexans of genus *Cephaloidophora* or ciliates of genus *Isochona*). Furthermore, measures of codon usage suggest that host- or habitat-specific selective conditions may contribute to IWaV278 evolution. These analyses lend further insight into the distribution of chimeric viruses and may provide a framework to better understand their evolutionary dynamics.

The presence of IWaV278 among intertidal isopods suggests that viruses with chimeric genomes may be more common among non-model aquatic organisms than previously suggested. Therefore, recombination and gene acquisition between small, circular ssDNA viruses and divergent viral taxa likely influences the genetic diversity, biogeographic range, and phylogenomic relationship among viruses in aquatic ecosystems. While phylogenetic and codon usage analyses of IWaV278 expand the known genetic diversity of the Cruciviridae, the origin and conditions of their ongoing evolution remains speculative. One major deficit in the study of chimeric viral genomes is the identity of their cellular hosts. This study provides the first evidence of a potential relationship between protozoan crustacean epibionts (apicomplexans and ciliates) and a chimeric genotype, indicating that unicellular eukaryotes may be responsible for the propagation and dissemination of chimeric viruses. Unicellular organisms occupy functionally critical niches in aquatic ecosystems as primary producers, metazoan parasites, heterotrophic bacterial consumers, and other ecologically-relevant members. Infection by chimeric viruses may influence unicellular eukaryote contributions to microbial nutrient cycling or net community structure, and further inquiry is essential to determine if the Cruciviridae have a demonstrable impact on unicellular eukaryote biology and ecology. 

## Supplementary Materials

The following are available online at www.mdpi.com/1999-4915/9/12/361/s1.
