# Supplementary material for: Distribution and Inferred Evolutionary Characteristics of a Chimeric ssDNA Virus Associated with Intertidal Marine Isopods
Source: Viruses. 2017 Nov 26;9(12):361. doi: 10.3390/v9120361 (PMC5744136; doi:10.3390/v9120361)
Supplement: Supplementary file 1 [file viruses-09-00361-s001.zip › 6.viruses-241354 suppl english/viruses-241354-supplementary (english edits)/Viruses_SupplementaryMaterial/Viruses_SupplementaryFigures (english edits).docx]

**Supplementary Figures**

| Metavirome Parameters | *Idotea (Pentidotea) resecata* | *Idotea (Pentidotea) wosnesenskii* | *Gnorimosphae-roma oregonensis* | Total |
| --- | --- | --- | --- | --- |
| Family | *Idoteidae* | *Idoteidae* | *Sphaeromatidae* | - |
| Collection location | Catalina Island, CA | Port Townsend, WA &  Ketchikan, AK | Port Townsend, WA | - |
| Total number of reads | 2,846,392 | 3,815,868 | 3,955,194 | 10,617,454 |
| Reads after trimming | 2,828,994 | 3,782,628 | 3,927,522 | 10,539,144 |
| N50 | 1,236 | 1,246 | 1,448 | 1,310 (Ave) |
| Contig length range (nt) | 386-29,372 | 363-39,847 | 362-18,908 | 362-39,847 |
| Mean contig length (nt) | 1,179 | 1,202 | 1,289 | 1,223 (Ave) |
| Total number of contigs | 6,051 | 12,112 | 20,911 | 39,074 |
| Annotated contigs | 45.9% | 51.4% | 48.7% | 48.7% (Ave) |
| Contigs homologous to metazoan associated CRESS-DNA viruses | 2 | 12 | 15 | 29 |
| CRESS-DNA virus contigs with putative Rep | 0 | 4 | 4 | 8 |
| Reads recruited to putative CRESS-DNA virus contigs | 55 | 3,873 | 7,709 | 11,637 |

**Table S1.** (**A**) Summary of metavirome statistics. Isopods were collected from Catalina Island, CA (33°26’42.0”N 118°29’02.4”W), Port Townsend, WA (48°08’31.2”N 122°46’55.2”W), and Ketchikan, AK (55°26’56.4’N 131°49’51.6”W) and prepared for metaviromic sequencing; 2×250 paired-end reads were generated via Illumina MiSeq and processed for barcode exclusion, length, and quality prior to de novo assembly on CLC Workbench v.8.5.1. N50 refers to contig length where 50% of assembled bases are within contigs of equal/greater length. Resulting contiguous sequences (contigs) and associated open reading frames (ORFs) were annotated via BLASTx (34). Read recruitment is reported as the absolute number of reads mapped to CRESS-DNA virus-like contigs with >80% identity over >50% of read length. Accession numbers: SAMN07716012-SAMN07716014 (BioProject PRJNA412272).

| **Target** | **Sequence (5’ -> 3’)** | |
| --- | --- | --- |
| IWaV278-*rep*  quantitation (qPCR) | Standard | ATGGCACAGAGAGGCTATGACACTGGTGCTGACCTCTCCCGTTTCGTGGGCTTCGGCGAATATAAAGGTGCC |
|  | Probe | [FAM] TGCTGACCTCTCCCGTTTCGTGG [TAMRA] |
|  | L-primer | TGGCACAGAGAGGCTATG |
|  | R-primer | GCACCTTTATATTCGCCGAAGC |
|  | LLOD ($\bar{x}$) | Ct of 37.96 corresponds to 39.3 amplicon copies |
| Thermocycling parameters (qPCR) include: 1 cycle at 95 °C x 5 min, followed by 60 cycles of 95 °C for 30s and 60 °C for 30s | | |
| IWaV278 genome completion | L-primer (outward bound) | CGACTTGCCAGCTCCCATT |
|  | R-primer  (outward bound) | GTGGACAGGTACCAAACAATTCG |
| Thermocycling parameters (PCR) include: 1 cycle 95°C for 2 min, 35 cycles of 30 s at 95°C, 60 s at 59°C and 60 s at 72°C each, and final extension for 6 min at 72°C | | |
| *Idotea* epibiont identity | L-primer (EU347F) | AGGGTTCGATTCCGGAGA |
|  | R-primer  (EU929R) | TTGGCAAATGCTTTCGC |
| Thermocycling parameters (PCR) include: 1 cycle 95°C for 2 min, 30 cycles of 30s at 95°C, 60 s at 55°C and 60 s at 72°C each, and final extension for 6 min at 72°C. Universal primers were derived from the European ribosomal RNA database. | | |

**Table S2.** (**B)** Primer/probe sequences and reaction parameters for qPCR and PCR quantitation of IWaV278 and putative *Idotea* *wosnesenskii* epibionts. All PCR and qPCR reactions were 25 µL. qPCR reactions included SsoAdvanced^TM^ Universal Probes Supermix (Bio-Rad Laboratories, Hercules, CA, USA) with 2 μM primer/probe oligo (Eurofins Scientific, Luxembourg City, Luxembourg) and 2 µL of template or external standard per reaction. LLOD specifies the average lower limit of detection (Ct) across all runs and the corresponding amplicon copy number. Samples with Ct values > LLOD were designated “no detection” (negative). Quantities were calculated per StepOnePlus software v.2.3 (Foster City, CA, USA) and standardized to extraction, elution, reaction dilution volumes, and isopod wet weight.

| **Contig Name** | **Best BLASTx hit** | **BLASTx e-value** | **Length (nt)** | **Coverage (ave)** |
| --- | --- | --- | --- | --- |
| GOaV1107 | *Phytophthora parasitica* virus isolate 1 - ORF3 (ref.NC_027197.1) | 2x10^-17^ | 1425 | 9.71 |
| GOaV1231 | Simian torque teno virus isolate VGA00123.2 - ORF1 gene (gb.KP296812.1) | 8x10^-6^ | 3572 | 21.52 |
| GOaV12856 | Simian torque teno virus isolate VWP00522.10 - ORF1 (gb.KP296842.1) | 3x10^-6^ | 1332 | 8.8 |
| GOaV15959 | Simian torque teno virus isolate VWP00516 - ORF1 (gb.KP296840.1) | 8x10^-6^ | 877 | 2.8 |
| GOaV16643 | Simian torque teno virus isolate VWP00457.4 - ORF1 (gb.KP296837.1) | 1x10^-7^ | 1019 | 3.37 |
| GOaV177 | Circovirus-like genome DCCV-3 (ref.NC_030467.1) | 4x10^-7^ | 628 | 9.08 |
| GOaV2188 | Uncultured marine virus clone SOG04070 (gb.JX904144.1) | 1x10^-9^ | 987 | 269.01 |
| GOaV2702 | Avon-Heathcote estuary associated bacilladnavirus (ref.NC_033744.1) | 1x10^-85^ | 4625 | 20.15 |
| GOaV2490 | Mosquito VEM virus SDRBAJ (gb.HQ335087.1) | 3x10^-7^ | 1112 | 254.76 |
| GOaV410 | Porcine adenovirus 3 (dbj.AB026117.1) | 6x10^-7^ | 7414 | 108.67 |
| GOaV4409 | Bat circovirus isolate BtRh-CV-7/Tibet2013 (gb.KJ641738.1) | 6x10^-55^ | 1906 | 39.41 |
| GOaV4852 | Odonata-associated circular virus-5 isolate OdasCV-5-US-1683LM1-12 (gb.KM598410.1) | 1x10^-7^ | 790 | 7.67 |
| GOaV5631 | Simian torque teno virus isolate VWP00522.9 - ORF1 (gb.KP296849.1) | 5x10^-6^ | 965 | 3.14 |
| GOaV7779 | Porcine adenovirus 3 (dbj.AB026117.1) | 1x10^-6^ | 596 | 2.83 |
| GOaV8926 | Uncultured marine virus clone SOG04106 (gb.JX904147.1) | 1x10^-52^ | 2060 | 26.09 |
| IRaV3058 | Simian torque teno virus isolate VWP00522.9 - ORF1 (gb.KP296849.1) | 3x10^-6^ | 1075 | 7.83 |
| IRaV3963 | Porcine adenovirus 3 (dbj.AB026117.1) | 1x10^-6^ | 867 | 3 |
| IWaV2030 | *Armadillidium nasatum* endogenous virus circovirus 46 – *Rep* (gb.KT714015.1) | 1x10^-7^ | 806 | 56 |
| IWaV278 | *Meles meles* circovirus-like virus (gb.JQ085285.1) | 2x10^-17^ | 3478 | 154.66 |
| IWaV2938 | Avon-Heathcote estuary associated bacilladnavirus (ref.NC_033744.1) | 9x10^-89^ | 1307 | 4.48 |
| IWaV3615 | Uncultured marine virus clone SOG04070 (gb.JX904144.1) | 1x10^-9^ | 985 | 39 |
| IWaV3812 | Odonata-associated circular virus-19 isolate OdasCV-19-US-1604SC1-12 (gb.KM598405.1) | 4x10^-6^ | 1113 | 14.94 |
| IWaV3868 | Circovirus-like genome DHCV-1 (ref.NC_030471.1) | 1x10^-8^ | 638 | 4.93 |
| IWaV6716 | Simian torque teno virus isolate VWP00516 - ORF1 (gb.KP296840.1) | 8x10^-6^ | 900 | 2.86 |
| IWaV7487 | *Armadillidium nasatum* endogenous virus circovirus 46- *Rep* (gb.KT714015.1) | 1x10^-7^ | 605 | 5 |
| IWaV7550 | Uncultured marine virus clone SI03513 (gb.JX904541.1) | 5x10^-11^ | 1588 | 4.1 |
| IWaV7922 | *Armadillidium nasatum* endogenous virus circovirus 46 - *Rep*(gb.KT714015.1) | 2x10^-7^ | 969 | 2.21 |
| IWaV872 | Uncultured marine virus clone SI00898 (gb.JX904478.1) | 1x10^-86^ | 2717 | 62.38 |
| IWaV9394 | Uncultured marine virus clone SI01813 (gb.JX904523.1) | 4x10^-11^ | 943 | 2.71 |

**Table S3.** Annotation of contigs sharing homology to metazoan-associated CRESS-DNA virus genomes. Contig abbreviations reflect associated isopod libraries (GOaV - *Gnorimosphaeroma oregonensis* associated virus; IRaV: *Idotea* *resecata;* IWaV: *Idotea* *wosnesenskii* associated virus) and identifying contig numbers (accession numbers: MG023125–MG023138). Contigs were annotated via BLASTx against the non-redundant (nr) database. Coverage was determined by absolute read recruitment (80% identity over 50% of read length) standardized by contig length (nt). Contigs represent both partial and complete (i.e., circularized) putative CRESS-DNA virus genomes.

**
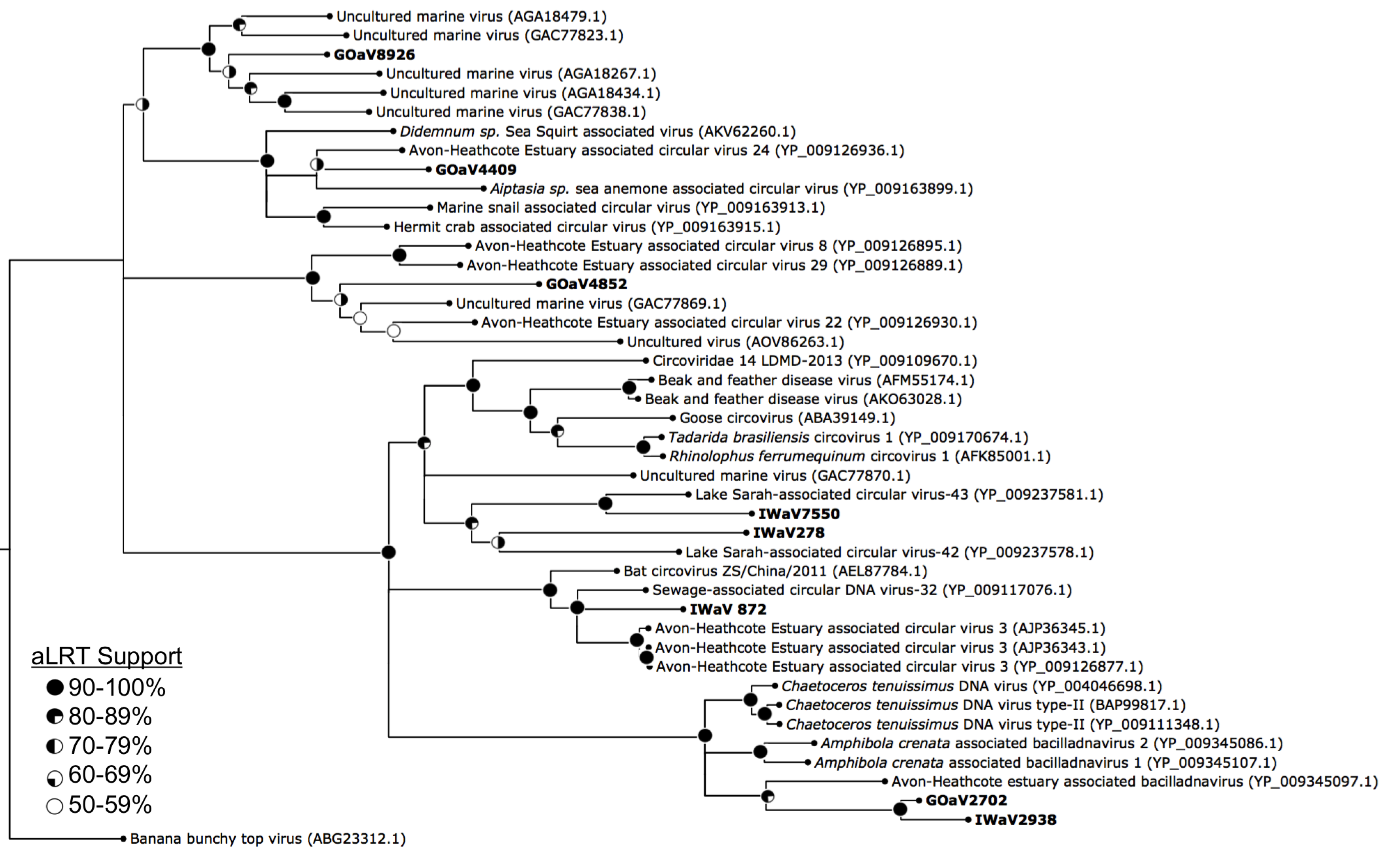
**

**Figure S1.** Maximum likelihood phylogeny of putative Rep ORFs of partial and complete contigs homologous to metazoan-associated CRESS-DNA viruses. Terminal nodes indicate Rep ORFs and associated top five best BLASTx hits (e-value < 10^–5^). Sequences were aligned in MUSCLE and manually masked (429 AA). Internal nodes represent SH-like aLRT branch support (model: Blosum62 +G+I+F).

| **ORF Name** | **Best BLASTx hit** | **BLASTx e-value** | **Length (nt)** | **%GC** | **GC1** | **GC2** | **GC3** | **CpG** | **ENC (20-61)** |
| --- | --- | --- | --- | --- | --- | --- | --- | --- | --- |
| IWaV29380*rep* | *Rep* - Avon-Heathcote estuary associated bacilladnavirus (YP_009345097.1) | 2x10^-81^ | 1068 | 42.0 | 47.8 | 38.5 | 39.9 | 0.9 | 59 |
| IWaV7550*rep* | *Rep -* Lake Sarah-associated circular virus-43 (YP_009237581.1) | 4x10^-36^ | 468 | 62.0 | 59.6 | 42.3 | 84.0 | 1.4 | 37 |
| IWaV872*rep* | *Rep* - Avon-Heathcote Estuary associated circular virus 3 (AJP36345.1) | 4x10^-106^ | 903 | 51.4 | 54.8 | 44.9 | 54.5 | 1.3 | 57 |
| IWaV278*rep* | *Rep* - Tadarida brasiliensis circovirus 1 (YP_009170674.1) | 5x10^-28^ | 1245 | 52.8 | 60.3 | 39.5 | 58.8 | 1.2 | 59 |
| GOaV4409*rep* | *Rep* - Marine snail associated circular virus (YP_009163913.1) | 1x10^-40^ | 306 | 44.4 | 50.0 | 28.4 | 54.9 | 1.1 | 41 |
| GOaV2702*rep* | *Rep* - Avon-Heathcote estuary associated bacilladnavirus (YP_009345097.1) | 2x10^-93^ | 1290 | 42.7 | 47.7 | 39.3 | 41.2 | 0.8 | 59 |
| GOaV8926*rep* | Hypothetical protein - Uncultured marine virus (AGA18479.1) | 4x10^-64^ | 825 | 39.5 | 39.3 | 36.4 | 42.9 | 0.6 | 57 |
| GOaV4852*rep* | *Rep -* Avon-Heathcote Estuary associated circular virus 8 (YP_009126895.1) | 8x10^-19^ | 447 | 43.0 | 50.3 | 34.2 | 44.3 | 0.6 | 45 |

**Table S4.** Description of CRESS-DNA virus Rep*-*like ORFs associated with temperate isopods. ORFs were called via GetORF and annotated via BLASTx (e-value < 1x10^–5^). %GC was determined across the complete ORF, and at synonymous and non-synonymous codon positions (GC1, GC2, and GC3). CpG refers to the observed frequency of CG dinucleotides standardized by the expected frequency (assuming equal distribution of dinucleotides across the ORF). ENC indicates the total effective number of codons (20 < n < 60), revealing codon specificity/preference among Rep ORFs.


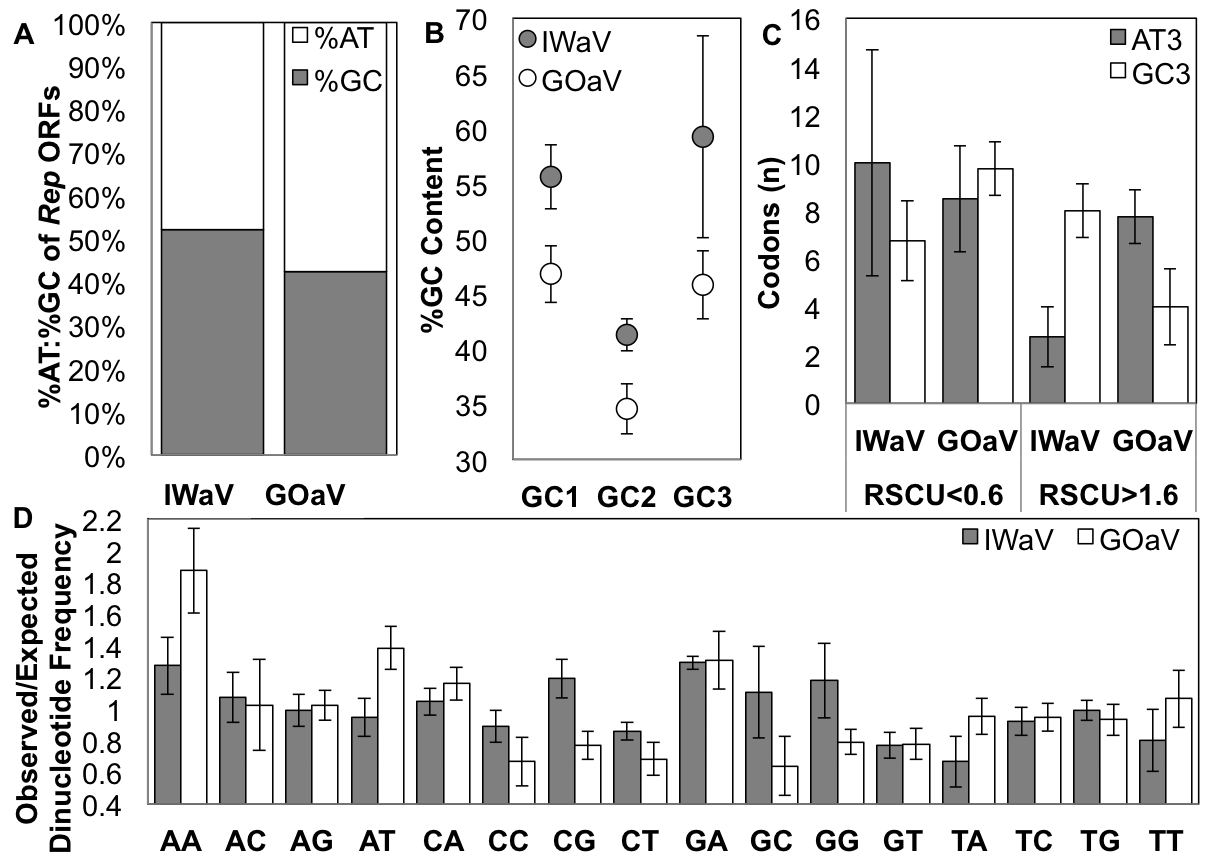


**Figure S2.** Graphical representation of nucleotide composition and codon usage bias among putative Rep ORFs associated with temperate isopods. (**A**) Average nucleotide composition, (**B**) average ±1 SE %GC content at codon positions GC1, GC2, and GC3; (**C**) average number ±1 SE of preferred (overrepresented, RSCU > 1.6) or underrepresented (RSCU < 0.6) codons ending with A/T or G/C; and (**D**) distribution of dinucleotide frequency (observed/expected frequency; assuming equal expected nucleotide frequency) across Rep ORFs associated with *I. wosnesenskii* (IWaV) *and G. oregonensis* (GOaV).


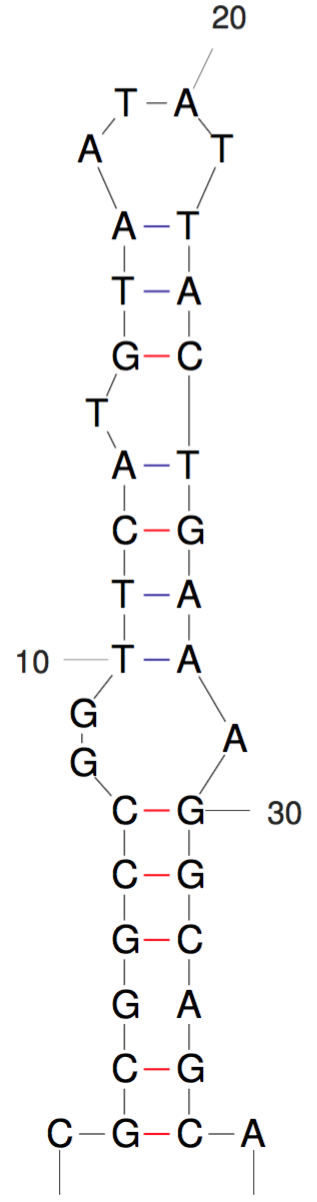


**Figure S3.** Stem loop structure of IWaV278 origin of replication. Predicted architecture of the stem loop (ΔG = –6.9 kcal/mol) located at the ori of IWaV278, predicted and visualized via Mfold. This structure comprises a nonanucleotide motif (TAATATTAC) canonical to CRESS-DNA viruses.


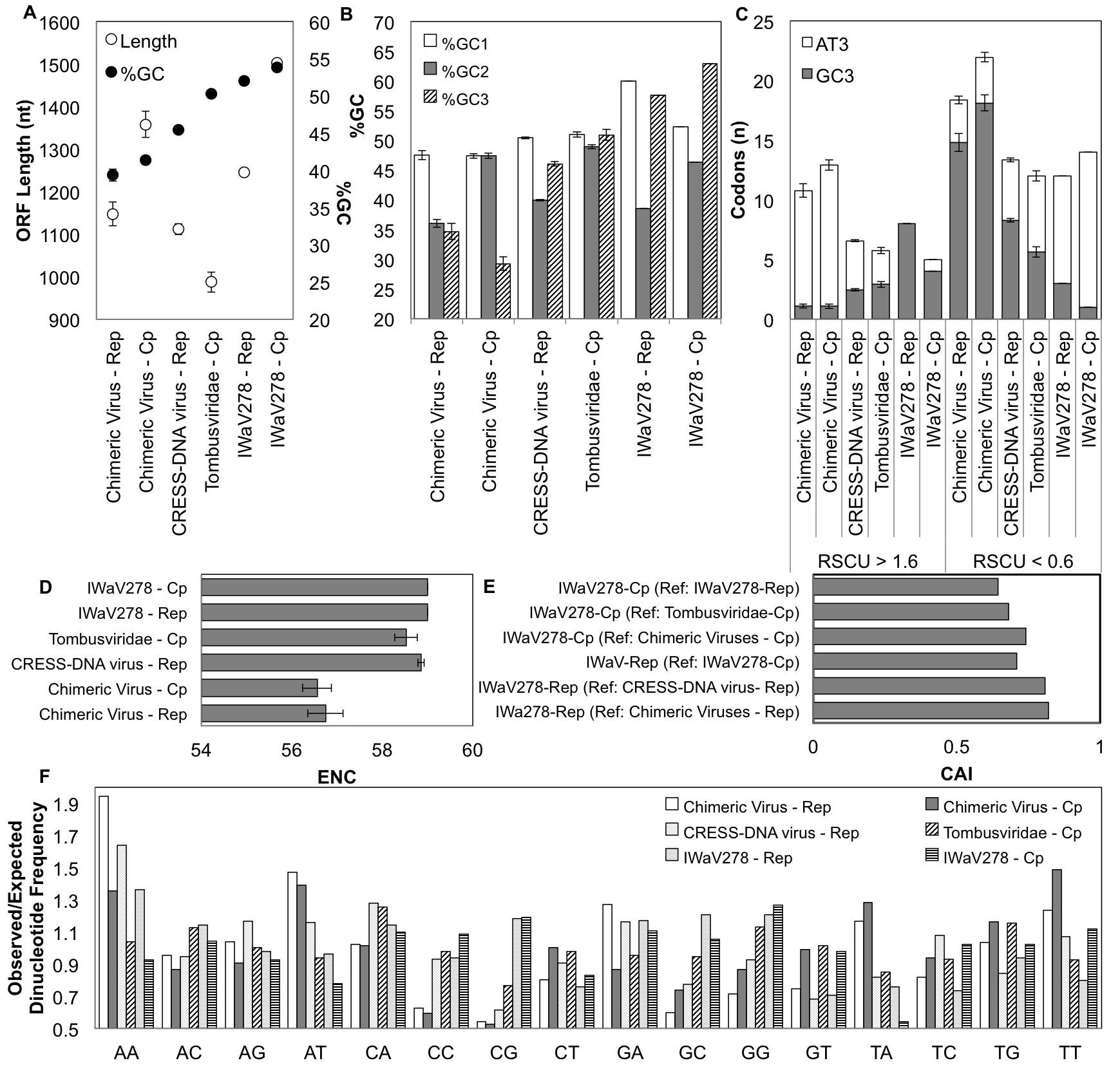


**Figure S4.** Codon usage patterns of IWaV278 ORFs relative to chimeric and non-chimeric genes: (**A**) Average ±1 SE length and total ORF %GC content; (**B**) average ±1 SE %GC content at synonymous (GC1 and GC2) and non-synonymous (GC3) codon positions depicting the degree of preference for G/C-terminating codons, (**C**) RSCU: average ±1 SE relative synonymous codon usage indicating over- or under-utilization (RSCU > 1.6 or RSCU < 0.6, respectively) of A/T- or G/C-terminating codons, as determined via comparison of observed codon frequency relative to expected codon frequency; (**D**) ENC: effective number of codons, denoting the average number of codons (±1 SE) utilized to express representative ORFs; (**E**) CAI: codon adaptation index indicating the measure of relatedness of IWaV278 ORFs relative to a set of reference genes (chimeric or non-chimeric ORFs); and (**F**) dinucleotide frequency delineating CpG content of *rep* and *cp* ORFs from chimeric viruses.

**
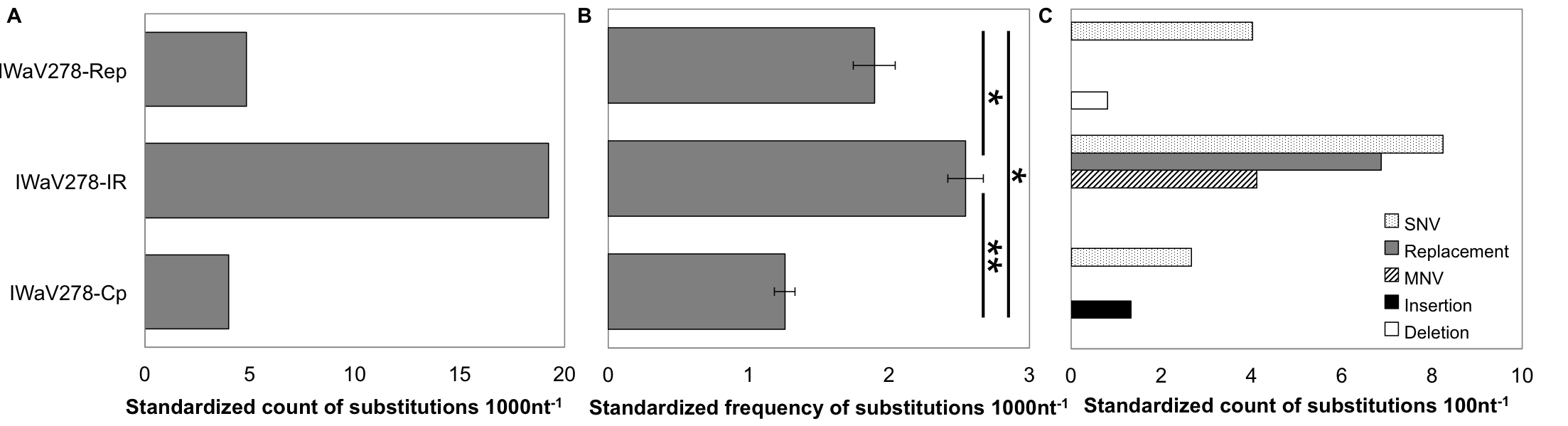
**

**Figure S5.** Position (**A**), frequency (**B**), and type (**C**) of single nucleotide variants within IWaV278. Variants were identified and classified by type via a multinomial model for low frequency variant calling, CLC Genomics Workbench v.8.5.1, corrected for ORF/intergenic region length (nt), and multiplied by 1000 to achieve standardized quantity (A), average frequency (±1 SE; B), or type (C) of substitution per thousand nucleotides (asterisk indicates significance, paired *t*-test: * *p <* 0.01, ***p <* 1 × 10^-7^).


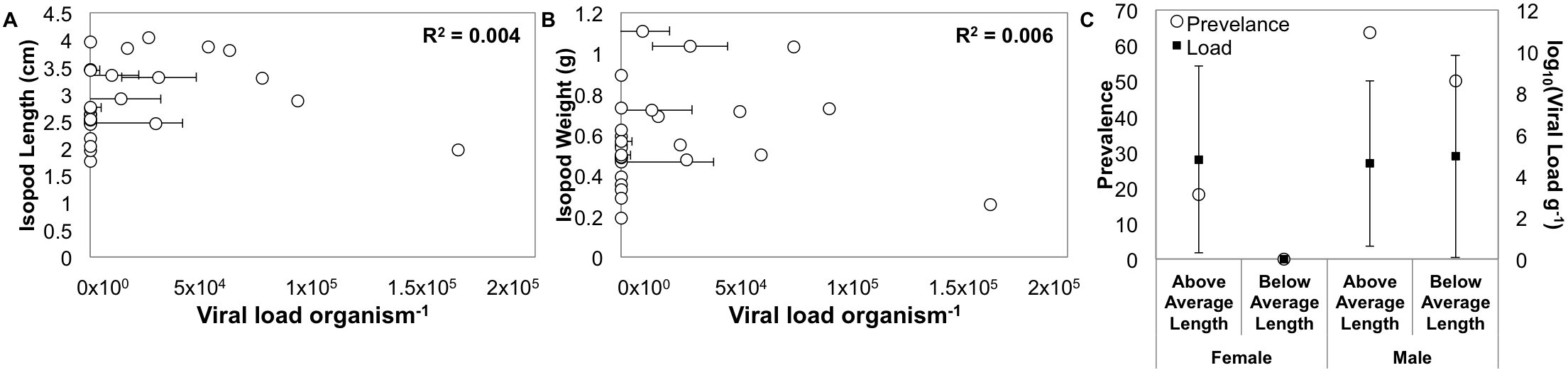


**Figure S6.** Quantitation of IWaV278 in male and female *I. wosnesenskii*. (**A**) Prevalence and (**B**) average load g^–1^ (±1 SE) of IWaV278 did not vary by organism length or weight when grouped by sex (**C**).

**
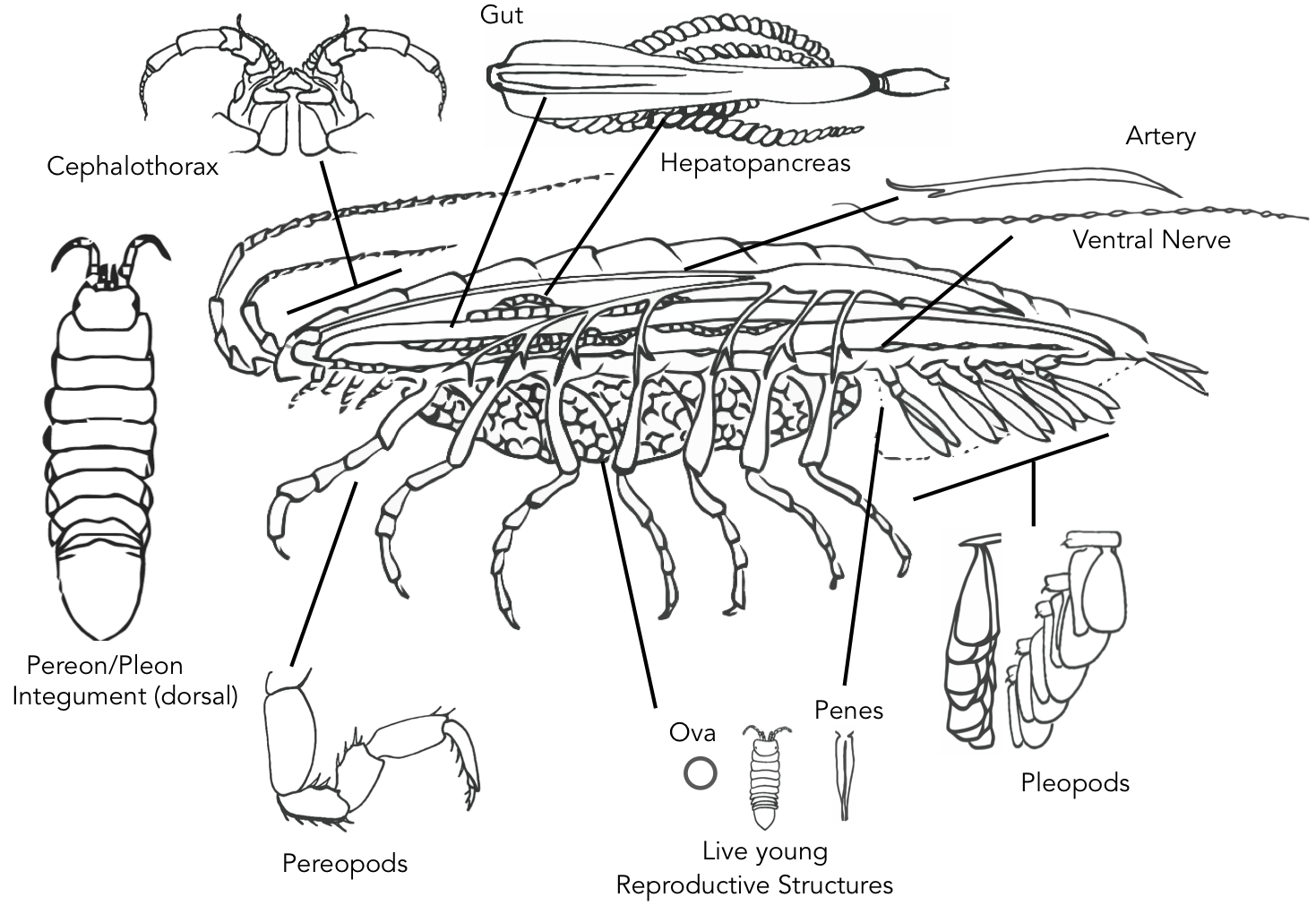
**

**Figure S7.** Diagram of *I. wosnesenskii* dissections. Depiction of major isopod organ systems isolated for qPCR detection.
