# Supplementary material for: Distribution and Inferred Evolutionary Characteristics of a Chimeric ssDNA Virus Associated with Intertidal Marine Isopods
Source: Viruses. 2017 Nov 26;9(12):361. doi: 10.3390/v9120361 (PMC5744136; doi:10.3390/v9120361)
Supplement: Supplementary file 1 [file viruses-09-00361-s001.zip › 6.viruses-241354 suppl english/viruses-241354-supplementary (english edits)/Viruses_SupplementaryMaterial/Viruses_SupplementaryFigures (english edits).pdf]

Supplementary Figures

**Metavirome Parameters**	***Idotea (Pentidotea) resecata***	***Idotea (Pentidotea) wosnesenskii***	***Gnorimosphae-roma oregonensis***	**Total**
Family	*Idoteidae*	*Idoteidae*	*Sphaeromatidae*	-
Collection location	Catalina Island, CA	Port Townsend, WA & Ketchikan, AK	Port Townsend, WA	-
Total number of reads	2,846,392	3,815,868	3,955,194	10,617,454
Reads after trimming	2,828,994	3,782,628	3,927,522	10,539,144
N50	1,236	1,246	1,448	1,310 (Ave)
Contig length range (nt)	386-29,372	363-39,847	362-18,908	362-39,847
Mean contig length (nt)	1,179	1,202	1,289	1,223 (Ave)
Total number of contigs	6,051	12,112	20,911	39,074
Annotated contigs	45.9%	51.4%	48.7%	48.7% (Ave)
Contigs homologous to metazoan associated CRESS-DNA viruses	2	12	15	29
CRESS-DNA virus contigs with putative Rep	0	4	4	8
Reads recruited to putative CRESS-DNA virus contigs	55	3,873	7,709	11,637

Table S1. (**A**) Summary of metavirome statistics. Isopods were collected from Catalina Island, CA (33°26′42.0″N 118°29′02.4″W), Port Townsend, WA (48°08′31.2″N 122°46′55.2″W), and Ketchikan, AK (55°26′56.4′N 131°49′51.6″W) and prepared for metaviromic sequencing; 2×250 paired-end reads were generated via Illumina MiSeq and processed for barcode exclusion, length, and quality prior to de novo assembly on CLC Workbench v.8.5.1. N50 refers to contig length where 50% of assembled bases are within contigs of equal/greater length. Resulting contiguous sequences (contigs) and associated open reading frames (ORFs) were annotated via BLASTx (34). Read recruitment is reported as the absolute number of reads mapped to CRESS-DNA virus-like contigs with >80% identity over >50% of read length. Accession numbers: SAMN07716012-SAMN07716014 (BioProject PRJNA412272).

Target	Sequence (5' -> 3')	
IWaV278-*rep* quantitation (qPCR)	Standard	ATGGCACAGAGAGGCTATGACACTGGTGCTGACCTCTCCCGTTTCGTGGGCTTCGGCGAATATAAAGGTGCC
	Probe	[FAM] TGCTGACCTCTCCCGTTTCGTGG [TAMRA]
	L-primer	TGGCACAGAGAGGCTATG
	R-primer	GCACCTTTATATTCGCCGAAGC
	LLOD ($\bar{x}$)	Ct of 37.96 corresponds to 39.3 amplicon copies
Thermocycling parameters (qPCR) include: 1 cycle at 95 °C x 5 min, followed by 60 cycles of 95 °C for 30s and 60 °C for 30s		
IWaV278 genome completion	L-primer (outward bound)	CGACTTGCCAGCTCCCATT
	R-primer (outward bound)	GTGGACAGGTACCAAACAATTCG
Thermocycling parameters (PCR) include: 1 cycle 95°C for 2 min, 35 cycles of 30 s at 95°C, 60 s at 59°C and 60 s at 72°C each, and final extension for 6 min at 72°C		
Idotea epibiont identity	L-primer (EU347F)	AGGGTTCGATTCCGGAGA
	R-primer (EU929R)	TTGGCAAATGCTTTCGC
Thermocycling parameters (PCR) include: 1 cycle 95°C for 2 min, 30 cycles of 30s at 95°C, 60 s at 55°C and 60 s at 72°C each, and final extension for 6 min at 72°C. Universal primers were derived from the European ribosomal RNA database.		

Table S2. (**B)** Primer/probe sequences and reaction parameters for qPCR and PCR quantitation of IWaV278 and putative *Idotea wosnesenskii* epibionts. All PCR and qPCR reactions were 25 µL. qPCR reactions included SsoAdvanced™ Universal Probes Supermix (Bio-Rad Laboratories, Hercules, CA, USA) with 2 µM primer/probe oligo (Eurofins Scientific, Luxembourg City, Luxembourg) and 2 µL of template or external standard per reaction. LLOD specifies the average lower limit of detection (Ct) across all runs and the corresponding amplicon copy number. Samples with Ct values > LLOD were designated "no detection" (negative). Quantities were calculated per StepOnePlus software v.2.3 (Foster City, CA, USA) and standardized to extraction, elution, reaction dilution volumes, and isopod wet weight.

Contig Name	Best BLASTx hit	BLASTx e-value	Length (nt)	Coverage (ave)
GOaV1107	*Phytophthora parasitica* virus isolate 1 - ORF3 (ref.NC_027197.1)	$2x10^{-17}$	1425	9.71
GOaV1231	Simian torque teno virus isolate VGA00123.2 - ORF1 gene (gb.KP296812.1)	$8x10^{-6}$	3572	21.52
GOaV12856	Simian torque teno virus isolate VWP00522.10 - ORF1 (gb.KP296842.1)	$3x10^{-6}$	1332	8.8
GOaV15959	Simian torque teno virus isolate VWP00516 - ORF1 (gb.KP296840.1)	$8x10^{-6}$	877	2.8
GOaV16643	Simian torque teno virus isolate VWP00457.4 - ORF1 (gb.KP296837.1)	$1x10^{-7}$	1019	3.37
GOaV177	Circovirus-like genome DCCV-3 (ref.NC_030467.1)	$4x10^{-7}$	628	9.08
GOaV2188	Uncultured marine virus clone SOG04070 (gb.JX904144.1)	$1x10^{-9}$	987	269.01
GOaV2702	Avon-Heathcote estuary associated bacilladnavirus (ref.NC_033744.1)	$1x10^{-85}$	4625	20.15
GOaV2490	Mosquito VEM virus SDRBAJ (gb.HQ335087.1)	$3x10^{-7}$	1112	254.76
GOaV410	Porcine adenovirus 3 (dbj.AB026117.1)	$6x10^{-7}$	7414	108.67
GOaV4409	Bat circovirus isolate BtRh-CV-7/Tibet2013 (gb.KJ641738.1)	$6x10^{-55}$	1906	39.41
GOaV4852	Odonata-associated circular virus-5 isolate OdasCV-5-US-1683LM1-12 (gb.KM598410.1)	$1x10^{-7}$	790	7.67
GOaV5631	Simian torque teno virus isolate VWP00522.9 - ORF1 (gb.KP296849.1)	$5x10^{-6}$	965	3.14
GOaV7779	Porcine adenovirus 3 (dbj.AB026117.1)	$1x10^{-6}$	596	2.83
GOaV8926	Uncultured marine virus clone SOG04106 (gb.JX904147.1)	$1x10^{-52}$	2060	26.09
IRaV3058	Simian torque teno virus isolate VWP00522.9 - ORF1 (gb.KP296849.1)	$3x10^{-6}$	1075	7.83
IRaV3963	Porcine adenovirus 3 (dbj.AB026117.1)	$1x10^{-6}$	867	3
IWaV2030	*Armadillidium nasatum* endogenous virus circovirus 46 – *Rep* (gb.KT714015.1)	$1x10^{-7}$	806	56
IWaV278	*Meles meles* circovirus-like virus (gb.JQ085285.1)	$2x10^{-17}$	3478	154.66
IWaV2938	Avon-Heathcote estuary associated bacilladnavirus (ref.NC_033744.1)	$9x10^{-89}$	1307	4.48
IWaV3615	Uncultured marine virus clone SOG04070 (gb.JX904144.1)	$1x10^{-9}$	985	39
IWaV3812	Odonata-associated circular virus-19 isolate OdasCV-19-US-1604SC1-12 (gb.KM598405.1)	$4x10^{-6}$	1113	14.94
IWaV3868	Circovirus-like genome DHCV-1 (ref.NC_030471.1)	$1x10^{-8}$	638	4.93
IWaV6716	Simian torque teno virus isolate VWP00516 - ORF1 (gb.KP296840.1)	$8x10^{-6}$	900	2.86
IWaV7487	*Armadillidium nasatum* endogenous virus circovirus 46- *Rep* (gb.KT714015.1)	$1x10^{-7}$	605	5
IWaV7550	Uncultured marine virus clone SI03513 (gb.JX904541.1)	$5x10^{-11}$	1588	4.1
IWaV7922	*Armadillidium nasatum* endogenous virus circovirus 46 - *Rep*(gb.KT714015.1)	$2x10^{-7}$	969	2.21
IWaV872	Uncultured marine virus clone SI00898 (gb.JX904478.1)	$1x10^{-86}$	2717	62.38
IWaV9394	Uncultured marine virus clone SI01813 (gb.JX904523.1)	$4x10^{-11}$	943	2.71

Table S3. Annotation of contigs sharing homology to metazoan-associated CRESS-DNA virus genomes. Contig abbreviations reflect associated isopod libraries (GOaV - *Gnorimosphaeroma oregonensis* associated virus; IRaV: *Idotea resecata;* IWaV: *Idotea wosnesenskii* associated virus) and identifying contig numbers (accession numbers: MG023125–MG023138). Contigs were annotated via BLASTx against the non-redundant (nr) database. Coverage was determined by absolute read recruitment (80% identity over 50% of read length) standardized by contig length (nt). Contigs represent both partial and complete (i.e., circularized) putative CRESS-DNA virus genomes.

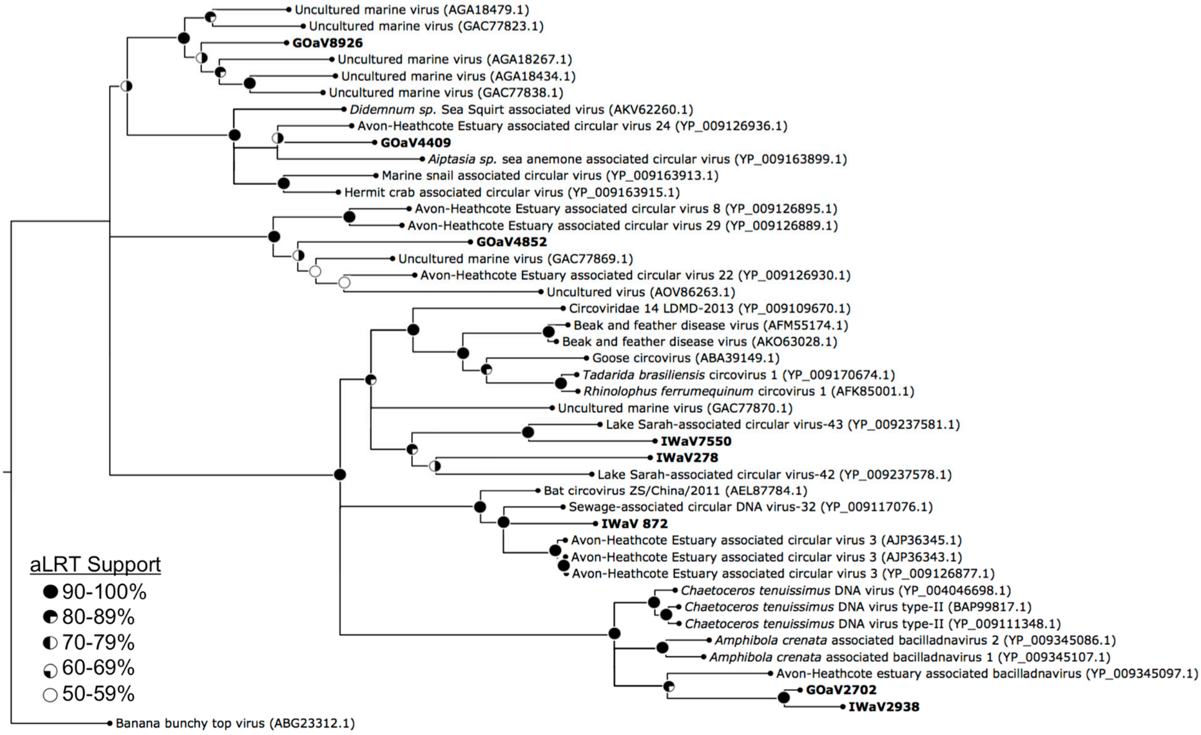

Figure S1. Maximum likelihood phylogeny of putative Rep ORFs of partial and complete contigs homologous to metazoan-associated CRESS-DNA viruses. Terminal nodes indicate Rep ORFs and associated top five best BLASTx hits (e-value < 10^{-5}). Sequences were aligned in MUSCLE and manually masked (429 AA). Internal nodes represent SH-like aLRT branch support (model: Blosum62 +G+I+F).

ORF Name	Best BLASTx hit	BLASTx e-value	Length (nt)	%GC	GC1	GC2	GC3	CpG	ENC (20-61)
IWaV29380*rep*	*Rep* - Avon-Heathcote estuary associated bacilladnavirus (YP_009345097.1)	$2x10^{-81}$	1068	42.0	47.8	38.5	39.9	0.9	59
IWaV7550*rep*	*Rep* - Lake Sarah-associated circular virus-43 (YP_009237581.1)	$4x10^{-36}$	468	62.0	59.6	42.3	84.0	1.4	37
IWaV872*rep*	*Rep* - Avon-Heathcote Estuary associated circular virus 3 (AJP36345.1)	$4x10^{-106}$	903	51.4	54.8	44.9	54.5	1.3	57
IWaV278*rep*	*Rep* - Tadarida brasiliensis circovirus 1 (YP_009170674.1)	$5x10^{-28}$	1245	52.8	60.3	39.5	58.8	1.2	59
GOaV4409*rep*	*Rep* - Marine snail associated circular virus (YP_009163913.1)	$1x10^{-40}$	306	44.4	50.0	28.4	54.9	1.1	41
GOaV2702*rep*	*Rep* - Avon-Heathcote estuary associated bacilladnavirus (YP_009345097.1)	$2x10^{-93}$	1290	42.7	47.7	39.3	41.2	0.8	59
GOaV8926*rep*	Hypothetical protein - Uncultured marine virus (AGA18479.1)	$4x10^{-64}$	825	39.5	39.3	36.4	42.9	0.6	57
GOaV4852*rep*	*Rep* - Avon-Heathcote Estuary associated circular virus 8 (YP_009126895.1)	$8x10^{-19}$	447	43.0	50.3	34.2	44.3	0.6	45

Table S4. Description of CRESS-DNA virus Rep-like ORFs associated with temperate isopods. ORFs were called via GetORF and annotated via BLASTx (e-value < $1x10^{-5}$). %GC was determined across the complete ORF, and at synonymous and non-synonymous codon positions (GC1, GC2, and GC3). CpG refers to the observed frequency of CG dinucleotides standardized by the expected frequency (assuming equal distribution of dinucleotides across the ORF). ENC indicates the total effective number of codons (20 < n < 60), revealing codon specificity/preference among Rep ORFs.

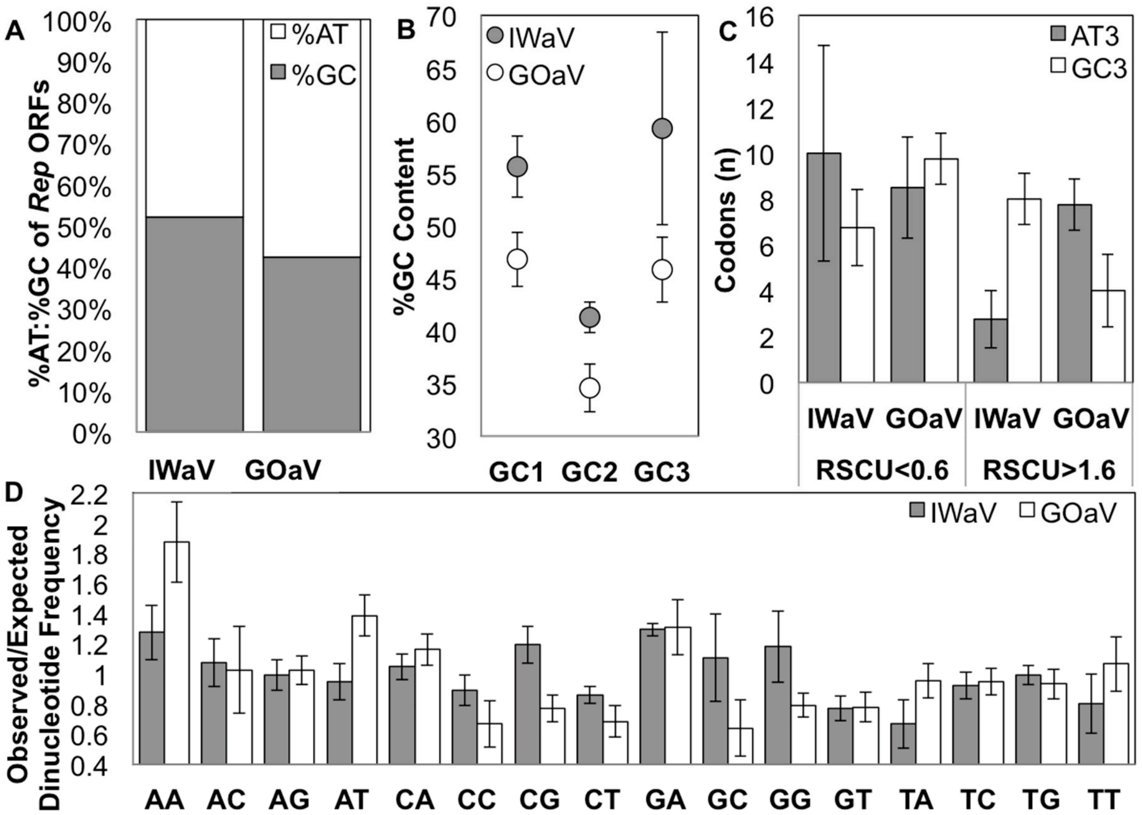

Figure S2. Graphical representation of nucleotide composition and codon usage bias among putative Rep ORFs associated with temperate isopods. (**A**) Average nucleotide composition, (**B**) average ±1 SE %GC content at codon positions GC1, GC2, and GC3; (**C**) average number ±1 SE of preferred (overrepresented, RSCU > 1.6) or underrepresented (RSCU < 0.6) codons ending with A/T or G/C; and (**D**) distribution of dinucleotide frequency (observed/expected frequency; assuming equal expected nucleotide frequency) across Rep ORFs associated with *I. wosnesenskii* (IWaV) *and G. oregonensis* (GOaV).

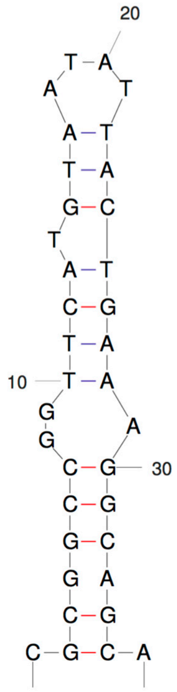

Figure S3. Stem loop structure of IWaV278 origin of replication. Predicted architecture of the stem loop (ΔG = –6.9 kcal/mol) located at the ori of IWaV278, predicted and visualized via Mfold. This structure comprises a nonanucleotide motif (TAATATTAC) canonical to CRESS-DNA viruses.

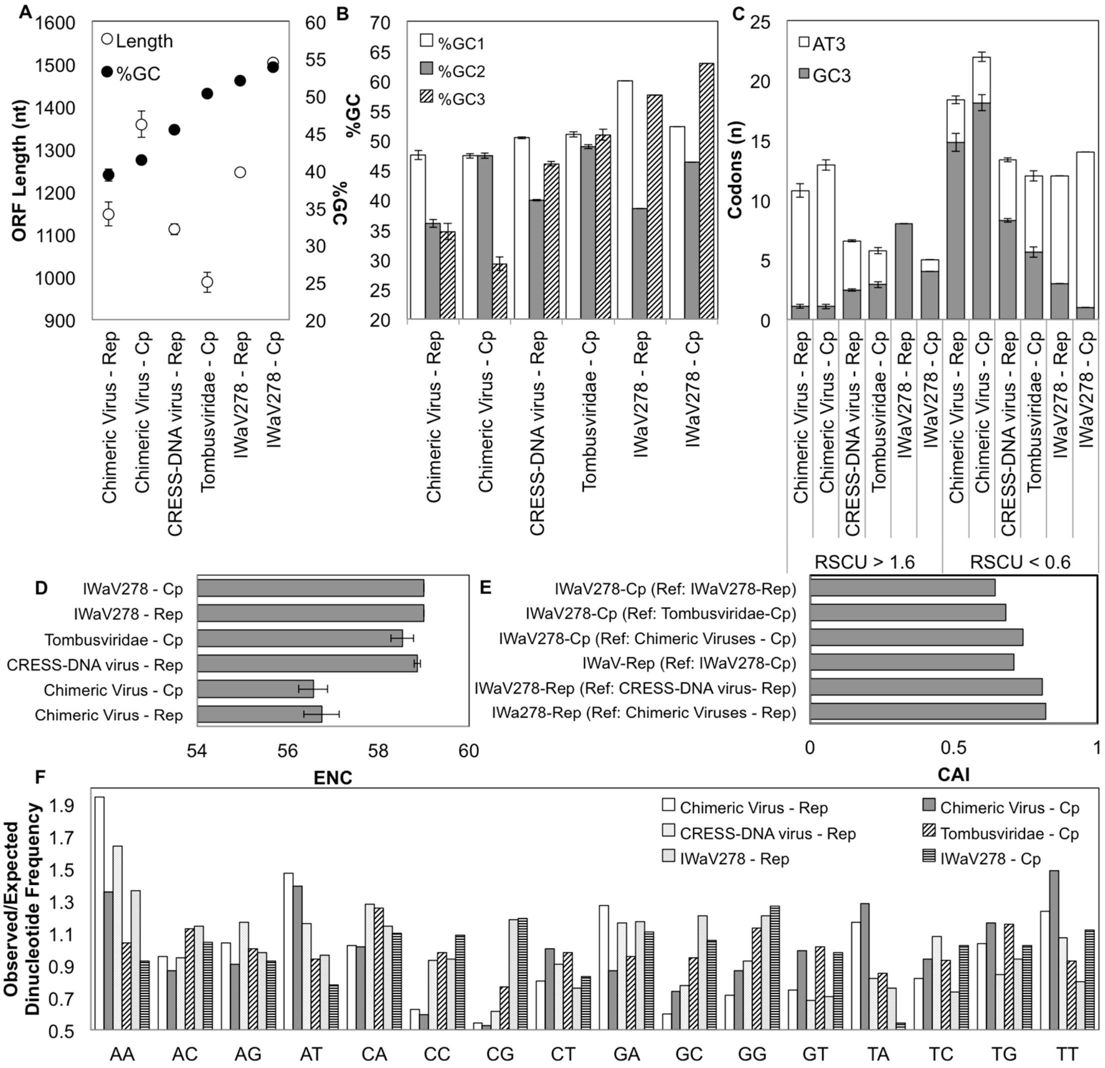

Figure S4. Codon usage patterns of IWaV278 ORFs relative to chimeric and non-chimeric genes: (**A**) Average ±1 SE length and total ORF %GC content; (**B**) average ±1 SE %GC content at synonymous (GC1 and GC2) and non-synonymous (GC3) codon positions depicting the degree of preference for G/C-terminating codons, (**C**) RSCU: average ±1 SE relative synonymous codon usage indicating over- or under-utilization (RSCU > 1.6 or RSCU < 0.6, respectively) of A/T- or G/C-terminating codons, as determined via comparison of observed codon frequency relative to expected codon frequency; (**D**) ENC: effective number of codons, denoting the average number of codons (±1 SE) utilized to express representative ORFs; (**E**) CAI: codon adaptation index indicating the measure of relatedness of IWaV278 ORFs relative to a set of reference genes (chimeric or non-chimeric ORFs); and (**F**) dinucleotide frequency delineating CpG content of *rep* and *cp* ORFs from chimeric viruses.

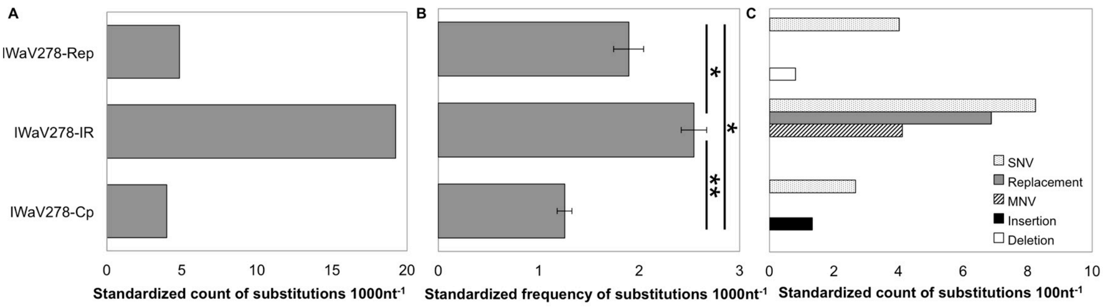

Figure S5. Position (**A**), frequency (**B**), and type (**C**) of single nucleotide variants within IWaV278. Variants were identified and classified by type via a multinomial model for low frequency variant calling, CLC Genomics Workbench v.8.5.1, corrected for ORF/intergenic region length (nt), and multiplied by 1000 to achieve standardized quantity (A), average frequency (±1 SE; B), or type (C) of substitution per thousand nucleotides (asterisk indicates significance, paired *t*-test: * $p < 0.01$, **$p < 1 \times 10^{-7}$).

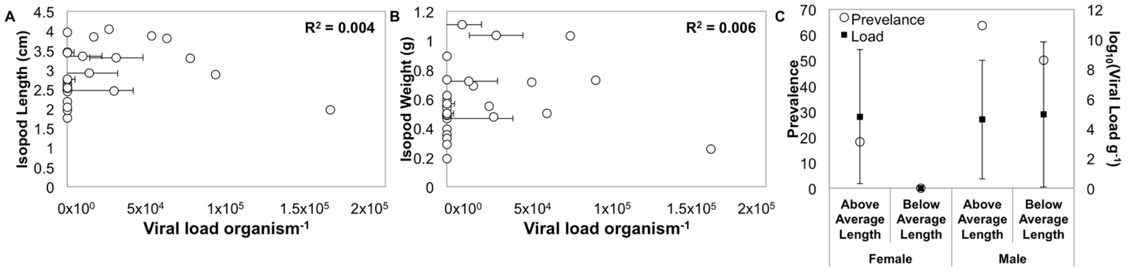

Figure S6. Quantitation of IWaV278 in male and female *I. wosnesenskii*. (**A**) Prevalence and (**B**) average load g^{-1} (±1 SE) of IWaV278 did not vary by organism length or weight when grouped by sex (**C**).

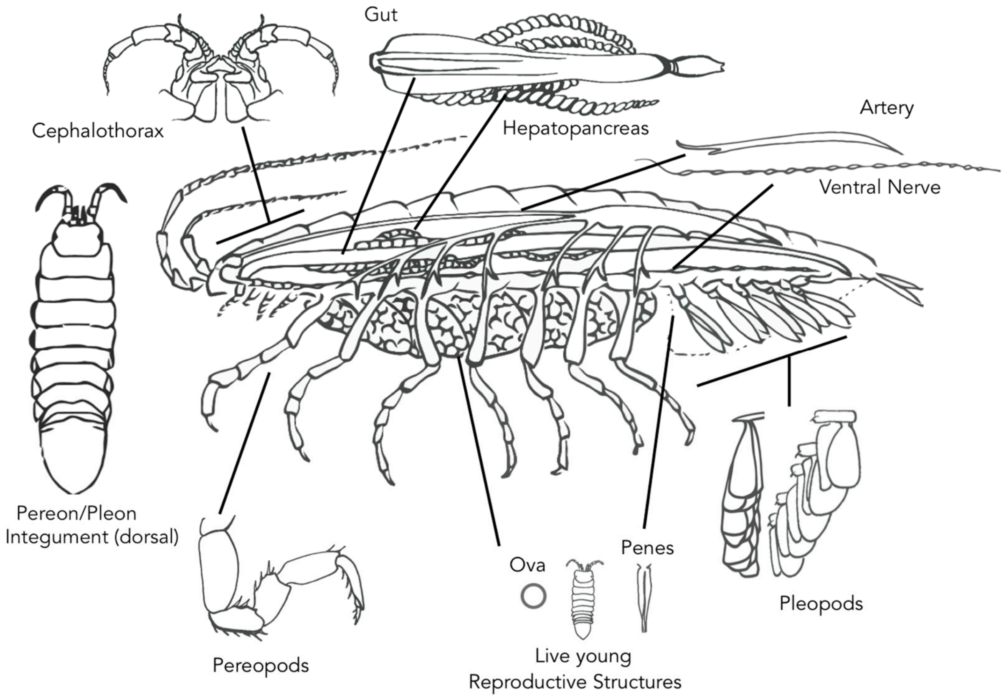

Figure S7. Diagram of *I. wosnesenskii* dissections. Depiction of major isopod organ systems isolated for qPCR detection.
